# Inflammation‐induced loss of CFTR‐expressing airway ionocytes in non‐eosinophilic asthma

**DOI:** 10.1111/resp.14833

**Published:** 2024-10-02

**Authors:** Ling Chen, Gabriela A. Hoefel, Prabuddha S. Pathinayake, Andrew Reid, Amber L. Pillar, Coady Kelly, HuiYing Tan, Ayesha Ali, Richard Y. Kim, Philip M. Hansbro, Steven L. Brody, Paul S. Foster, Jay C. Horvat, Carlos Riveros, Nikhil Awatade, Peter A. B. Wark, Gerard E. Kaiko

**Affiliations:** ^1^ School of Biomedical Sciences and Pharmacy University of Newcastle Newcastle New South Wales Australia; ^2^ Immune Health Program Hunter Medical Research Institute New Lambton Heights New South Wales Australia; ^3^ School of Medicine and Public Health University of Newcastle Newcastle New South Wales Australia; ^4^ School of Life Sciences University of Technology Sydney Sydney New South Wales Australia; ^5^ Centre for Inflammation Centenary Institute and University of Technology Sydney, Faculty of Science, School of Life Sciences Sydney New South Wales Australia; ^6^ Department of Medicine Washington University School of Medicine in St Louis St Louis Missouri USA; ^7^ Department of Respiratory and Sleep Medicine John Hunter Hospital New Lambton New South Wales Australia; ^8^ Department of Respiratory Medicine Alfred Health Melbourne Victoria Australia

**Keywords:** airway epithelium, CFTR, ionocytes, mucous, neutrophils, severe asthma, single cell RNA‐sequencing

## Abstract

**Background and Objective:**

Severe asthma is a heterogeneous disease with subtype classification according to dominant airway infiltrates, including eosinophilic (Type 2 high), or non‐eosinophilic asthma. Non‐eosinophilic asthma is further divided into paucigranulocytic or neutrophilic asthma characterized by elevated neutrophils, and mixed Type 1 and Type 17 cytokines in the airways. Severe non‐eosinophilic asthma has few effective treatments and many patients do not qualify for biologic therapies. The cystic fibrosis transmembrane conductance regulator (CFTR) is dysregulated in multiple respiratory diseases including cystic fibrosis and chronic obstructive pulmonary disease and has proven a valuable therapeutic target. We hypothesized that the CFTR may also play a role in non‐eosinophilic asthma.

**Methods:**

Patient‐derived human bronchial epithelial cells (hBECs) were isolated and differentiated at the air‐liquid interface. Single cell RNA‐sequencing (scRNAseq) was used to identify epithelial cell subtypes and transcriptional activity. Ion transport was investigated with Ussing chambers and immunofluorescent quantification of ionocyte abundance in human airway epithelial cells and murine models of asthma.

**Results:**

We identified that hBECs from patients with non‐eosinophilic asthma had reduced CFTR function, and did not differentiate into CFTR‐expressing ionocytes compared to those from eosinophilic asthma or healthy donors. Similarly, ionocytes were also diminished in the airways of a murine model of neutrophilic‐dominant but not eosinophilic asthma. Treatment of hBECs from healthy donors with a neutrophilic asthma‐like inflammatory cytokine mixture led to a reduction in ionocytes.

**Conclusion:**

Inflammation‐induced loss of CFTR‐expressing ionocytes in airway cells from non‐eosinophilic asthma may represent a key feature of disease pathogenesis and a novel drug target.

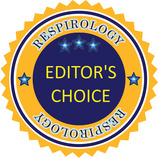

## Graphical abstract

We demonstrate reduced CFTR function, CFTR protein‐expressing cells and airway ionocytes in non‐eosinophilic severe asthma in the setting of a Type 1/Type 17 cytokine environment. This suggests a role for dysfunction in the CFTR pathway applicable to other chronic neutrophil‐dominant airways diseases, including subtypes of asthma.

**See related** editorial

## INTRODUCTION

Severe asthma impacts approximately 1 in 10 people diagnosed with asthma, and is responsible for the majority of the healthcare burden, hospitalizations and treatment cost.^1^ Patients with severe asthma are refractory to inhaled corticosteroids and the majority are characterized by eosinophilic airway inflammation (sputum or bronchoalveolar lavage [BAL]), with elevated exhaled nitric oxide (FeNO), and Type 2 gene signatures (IL‐4, ‐5 and ‐13). It is increasingly recognized that in severe asthma, airway inflammation among patients is heterogeneous, with many patients demonstrating non‐eosinophilic or Type 2 low airway inflammation.^2^, ^3^ Non‐eosinophilic asthma can be further sub‐divided into neutrophilic asthma patients with increased airway neutrophils and low eosinophils marked by elevated levels of Type 1 (IFN‐γ, TNF‐α) and Type 17 (IL‐17A, IL‐22) cytokines, or paucigranulocytic asthma patients with low levels of both eosinophils and neutrophils and a mixed inflammatory cytokine profile.^4^

Identification of severe asthma with Type 2 and eosinophilic characteristics refractory to inhaled corticosteroids is now a key part of the clinical assessment, defining those who will respond to monoclonal antibody therapy directed against IgE, IL‐5 and IL‐4/13.^1^ However, those with severe asthma without evidence of active type 2 inflammation or non‐eosinophilic asthma remain difficult‐to‐treat with a lack of effective treatment options.^1^ In particular, neutrophilic asthma has significant unmet need for effective therapeutic options. Interestingly, neutrophilic asthma bears many of the hallmark features of other chronic neutrophilic airway diseases such as chronic obstructive pulmonary disease (COPD) and cystic fibrosis (CF), including airway neutrophilia, higher bacterial colonization, goblet cell hyperplasia and Type 1 and 17 dominated immune signatures.^5^, ^6^, ^7^, ^8^, ^9^, ^10^ Given that COPD and CF have known defects in CFTR (Cystic fibrosis transmembrane conductance regulator) anion channel regulation and that even mild defects in this pathway (in the case of adult‐onset CF) are sufficient to induce chronic airway disease, we hypothesized that there may be an induced dysfunction in this pathway in severe non‐eosinophilic asthma. Furthermore, a case series report also correlated non‐eosinophilic asthma with abnormal nasal potential difference responses, a surrogate of CFTR function.^11^

In this study, we demonstrate that dysregulated ion channel transport through the CFTR and inflammation‐induced loss of ionocytes may play a role in the pathogenesis of non‐eosinophilic asthma and represents a novel therapeutic target in this difficult‐to‐treat disease.

## METHODS

Further details of Methods can be found in Appendix S1 in the Supporting Information, including scRNAseq computational data analysis, measurement and analysis of ciliary beat frequency and immunofluorescence analysis.

### Participants

All subjects were 18 years or older, non‐smokers or ex‐smokers with less than 5 pack years smoked. Any subject demonstrating acute respiratory or any other illness within the previous 6 weeks was excluded. Spirometry was performed according to ATS/ERS guidelines.^12^ Exhaled nitric oxide was measured using a NIOX Vero.^13^ Healthy control subjects had no history of lung disease, were non‐smokers with less than 5 pack years history of smoking and had normal spirometry.

Asthma was defined by; a physician's diagnosis, together with a bronchodilator response of ≥12% and ≥200 mL of FEV_1_ or documented airway hyper responsiveness defined by a 15% decline in FEV_1_ during indirect bronchial provocation test, respectively, or evidence of demonstrable peak flow variability of more than 15% between the two highest and two lowest peak expiratory flow readings for 28 days. Asthma severity was defined by GINA guidelines; all participants required GINA step 4 treatment,^14^ with at least 500 mcg of fluticasone propionate or equivalent daily along with a long‐acting beta agonist. Approval of this study was obtained from the Hunter New England Health human research ethics committee. Subjects and patients with severe asthma were recruited through the John Hunter Hospital clinics. Written informed consent was obtained from the study subjects.

### Cells obtained from human tissue

BAL fluid was processed and differential cells counts were performed as previously described.^15^ Inflammatory asthma subtypes were defined based on BAL cell counts as previously reported,^15^ and defined as (a) neutrophilic asthma when differential count was ≥60% neutrophils plus ≥0.99 × 10^6^ cells/mL total cell count (TCC) and <4% eosinophils; (b) eosinophilic asthma, ≥4% eosinophils and <60% neutrophils or (c) paucigranulocytic asthma, <4% eosinophils and <60% neutrophils. The clinical characteristics of the study subjects are shown in Table S1 in the Supporting Information.

Human bronchial epithelial cells (hBECs) were collected during clinical bronchoscopy by endobronchial brushings of the third–fourth branch as previously described.^16^ Human tracheal epithelial cells (hTECs) were isolated from excess surgical tissue of lungs donated for transplant at Washington University in St Louis as previously described^17^ and exempted from regulation as human subject research by the Washington University in St Louis Institutional Review Board.

### Murine asthma models induced by OVA, *Chlamydia muridarum* and *Alternaria alternata*

Mouse studies were performed in accordance with protocols for the care and use of animals approved by the Animal Ethics Committee of The University of Newcastle. Wild‐type BALB/c mice were sensitized to OVA (ovalbumin 50 μg, intraperitoneal [i.p.] injection, Sigma‐Aldrich) in Rehydragel® (1 mg [Reheis] in 200 μL PBS under isoflurane anaesthesia). Mice were subsequently challenged intranasally (i.n.) with OVA (10 μg/50 μL sterile saline) on d12–13 to induce airways disease. On d14 mice were given 50 μL SPG vehicle (sucrose‐phosphate‐glutamate buffer pH 7.5) i.n. and challenged with OVA again on d33–34 to exacerbate the airways disease (*n* = 10 mice).

*Chlamydia muridarum* (Cmu), a respiratory infection in mice mimicking *Chlamydia pneumoniae* in humans, was used to induce neutrophilic airway inflammation as previously described.^18^, ^19^ Cmu/OVA mice on d14 received Cmu (ATCC VR‐123) as 100 inclusion‐forming units in 50 μL SPG, i.n (*n* = 10 mice). Another set of wild‐type BALB/c mice were challenged i.n. with *Alternaria alternata* (AA; Greer) 5 μg in 50 μL PBS, three times per week, for 5 weeks (*n* = 7 mice). Control mice received either phosphate buffered saline or SPG buffered saline alone (*n* = 11 mice). Lungs were fixed in 4% paraformaldehyde (PFA) overnight, washed in 70% ethanol and then embedded in paraffin blocks for sectioning. BAL was collected and quantified by differential counts as previously described.^20^

### Air–liquid interface (ALI) culture of primary airway epithelial cells

Normal donor hTECs and healthy and asthma donor‐derived hBECs were cultured in BEGM (Lonza) supplemented with BSA 8 μg/mL, 1% fungizone and 2% penicillin/streptomycin in a six‐well plate. Cells were released by trypsin and seeded at 7 × 10^4^ cells per well in 6.5 mm Transwell with 0.4 μm pore polyester membrane inserts (Corning). Cells were grown at air liquid interface (ALI) culture as previously described.^21^ Briefly, cell membranes were submerged in BEGM/DMEM with supplements rhEGF (10 ng/mL) and retinoic acid (30 ng/mL) until confluency. ALI was established by removal of apical medium, and cells were cultured with basal BEGM/DMEM with supplements rhEGF (0.5 ng/mL) and retinoic acid (30 ng/mL). Cultures were maintained at ALI and experiments conducted at 30 days.

For cytokine experiments, cells were treated with a mixture of Type 1/Type 17 cytokines containing IFNγ (10 ng/mL), IL‐22 (10 ng/mL), IL‐17A (10 ng/mL) and TNFα (1 ng/mL) or these individual cytokines alone once per week for the final 2 weeks on d21 and d28 or IL‐13 alone (20 ng/mL) for the final 3 weeks. In some experiments, cells were treated with CFTR modulators (Selleckchem) at previously optimized doses: VX661 (6.67 μM), VX445 (6.67 μM) for 24 h and VX770 (10 μM) 1 h prior to Ussing measurements as well as in chamber during forskolin stimulation.

### Ussing chamber analysis of ion transport in airway epithelia

Ion transport properties in ALI cultured bronchial epithelial cells were studied by EasyMount Ussing Chamber Systems (VCC MC8 multichannel voltage/current clamp, Physiologic Instruments, Inc.). In brief, the apical and basolateral sides of the epithelium were perfused continuously with KRB buffer solution of the following composition: 115 mM NaCl, 2.4 mM K_2_HPO_4_, 0.4 mM KH_2_PO_4_, 1.2 mM CaCl_2_ dihydrate, 1.2 mM MgCl_2_ hexahydrate, 25 mM NaHCO_3_, 10 mM HEPES and 10 mM glucose, pH 7.4. The chambers were continuously gassed with 95% O_2_–5% CO_2_ and maintained at 37°C. The short‐circuit current (Isc) and transepithelial resistance (Rt) were determined under voltage‐clamp conditions. The Isc is a direct measure for the net movement of ions across the epithelium and recorded every 30 seconds. Data were acquired using Acquire and Analyse (version 2.3) software (Physiologic Instruments). The following specific compounds were added to the apical (A) and/or basolateral (B) bathing solutions in a standardized sequence. Cell membranes were equilibrated for 20 minutes in the presence of indomethacin (10 μM, A + B) to inhibit endogenous chloride secretion caused by prostaglandin synthesis. Amiloride (100 μM, A) was added to block amiloride‐sensitive sodium ion absorption. Forskolin (10 μM, A + B) and 3‐isobutyl‐1‐methylxanthine (IBMX) (100 μM, A + B) were added to activate cAMP‐dependent CFTR function with chloride and bicarbonate ion transport. Carbachol (100 μM, B) was added to stimulate cholinergic calcium ion transport. CFTRinh‐172 (25 μM, A + B) was added to inhibit CFTR‐dependent anion transport. Isc responses to forskolin and IBMX (∆Isc‐Fsk + IBMX) was used as an indicator of maximum CFTR function.

### Single‐cell RNA sequencing (scRNAseq) on airway epithelial cells

Airway epithelial cells were released by trypsin from Transwell membranes monitored under microscopy and processed using the 10XGenomics Chromium Next GEM v3.1 mRNA 3′ protocol as per manufacturer's instructions. Briefly, single cells were partitioned into gel beads in emulsion with cell lysis and reverse transcription of RNA introduced cell barcoding. This step was followed by PCR amplification, clean‐up and double‐sided size selection with SPRIselect reagent (Beckman Coulter), and adaptor ligation and sample indexing by PCR. The hTECs were assessed using the same protocol but with v3 chemistry. We aimed to recover a maximum of 10,000 cells per sample for hBEC experiments and 3000 cells per sample for hTEC experiments. hBEC samples were mixed at an equimolar ratio after indexing and sequenced with a Novaseq 6000 S1 flow cell followed by de‐multiplexing. All hBEC samples were isolated and sequenced simultaneously, avoiding the need for batch correction. The subjects chosen for the healthy control and asthma subtype groups for the scRNAseq study matched the clinical parameters as specified in Table S1 in the Supporting Information for total subjects. hTEC samples were sequenced separately.

### Pathway analysis

Multiple pathway analyses were performed using the gene lists derived from scRNAseq analysis as described. This included Ingenuity Pathways Analysis (IPA), ConsensusPathDB and ENRICHR^22^, ^23^ for Gene Ontology (GO) of biological and molecular processes, multi‐pathway over‐representation statistical analysis and Human Phenotype Ontology. The Library of Integrated Network‐Based Cellular Signatures (LINCS) L1000 dataset was used to investigate ligands known to regulate gene signatures similar to the ionocyte gene signature.

### Immunofluorescence

Transwell membranes containing hBECs were fixed using 4% PFA for 15 min at room temperature. For whole mount staining, membranes were embedded in HistoGel (Thermo Scientific) and labelled as previously described (see Supporting Information Appendix A for more detail).^21^

### Statistical analysis

Statistical analysis was performed by using the GraphPad Prism software (Version 9.0). Normality tests of data were conducted with the Shapiro–Wilk and D'Agostino–Pearson tests. Data that followed a normal distribution was tested using one‐way ANOVA to identify differences between two or more experimental groups with correction for multiple comparisons, and student unpaired or paired *t* tests were used where appropriate for comparisons of two groups. For data that was non‐parametric a Mann–Whitney *t* test or Kruskal–Wallis test was used as indicated in figure legends. All values are considered significant at a *p* value of less than 0.05.

## RESULTS

### CFTR function is diminished in bronchial epithelial cells from patients with severe non‐eosinophilic asthma

**FIGURE 1:**
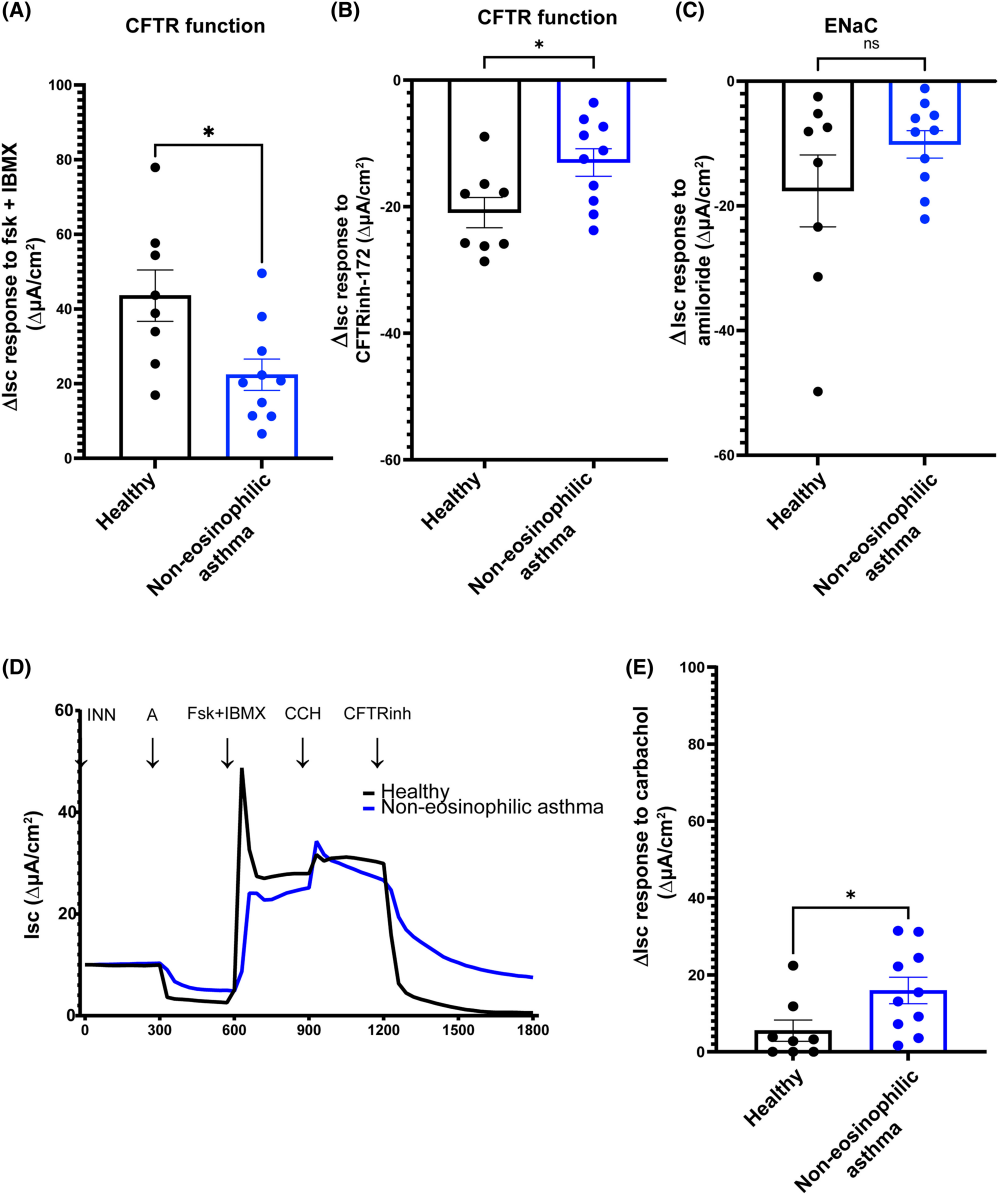
Ion transport measurements in healthy and non‐eosinophilic asthma human bronchial epithelial cells (hBECs) at air–liquid interface (ALI). The delta values of short circuit currents (∆Isc) for (A) CFTR‐Forskolin + 3‐isobutyl‐1‐methylxanthine (IBMX) stimulated, (B) CFTRinh‐172 inhibitor specifically blocking CFTR‐dependent activity, and (C) Amiloride‐sensitive ENaC currents for healthy (*n* = 8) and non‐eosinophilic asthma (*n* = 10) hBECs grown at ALI for 28 days. (D) Representative Isc responses of hBECs (circle line represents a healthy subject, and square line represents a non‐eosinophilic asthma subject) after sequentially stimulated with amiloride (100 μM), forskolin (10 μM) + IBMX (100 μM), carbachol (100 μM) and CFTRinh‐172 (25 μM). INN, indomethacin; A, amiloride; Fsk, forskolin; IBMX, 3‐isobutyl‐1‐methylxanthine; CCH, carbachol; CFTRinh, CFTRinh‐172 inhibitor. (E) ∆Isc for response to calcium activated carbachol channels for healthy (*n* = 8) and non‐eosinophilic asthma (*n* = 10) hBECs grown at ALI for 28 days. Each circle or square represents one individual subject. Error bars represent standard error of the mean (Mean ± SEM). Unpaired *t* test (A–C) or Mann–Whitney *t* test (E) was used to determine statistical significance, *p < 0.05, **p < 0.01.

Given the similar inflammatory cell profiles between severe non‐eosinophilic asthma with COPD and CF, we assessed whether CFTR function was altered in hBECs from patients with severe non‐eosinophilic asthma. Patient‐derived cells were differentiated under standard conditions at air‐liquid interface (ALI) and assessed by standard Ussing chamber ion transport measurements. Results revealed a significant reduction (~50%) in CFTR function and anion transport in non‐eosinophilic asthma hBECs (Figure 1A). This finding was confirmed by inhibition of the CFTR channel with the small molecule CFTR‐72 (Figure 1B). Amiloride stimulation, which inhibits the ENaC epithelial sodium ion channel, did not show a significant difference between non‐eosinophilic asthma and healthy controls (Figure 1C,D). We also observed an increase in carbachol stimulated ion transport in non‐eosinophilic asthma compared to healthy controls (Figure 1E). These data reveal a dysregulation of CFTR anion channel transport in differentiated airway epithelial cells from patients with non‐eosinophilic asthma. Given the importance of CFTR to anion transport, airway mucus dehydration and hypersecretion and homeostatic lung defences through mucociliary clearance, we sought to further investigate the cell and molecular basis of this funding in severe non‐eosinophilic asthma.

### scRNA‐seq reveals loss of CFTR‐expressing ionocytes in severe non‐eosinophilic asthma

To assess airway epithelial cell subtype and transcriptional differences between healthy and severe asthma subtypes we conducted scRNA‐seq analysis on patient‐derived ALI differentiated hBECs from health donors, severe eosinophilic asthma and severe non‐eosinophilic asthma, including neutrophilic and paucigranulocytic patients (characterized by BAL cell counts, *n* = 4 patients each) (Figure S1A in the Supporting Information). Cell subtypes were clustered using Seurat and known subtypes classified using classical airway epithelial markers with leading edge analysis (Tables S2 and S3 in the Supporting Information).

**FIGURE 2:**
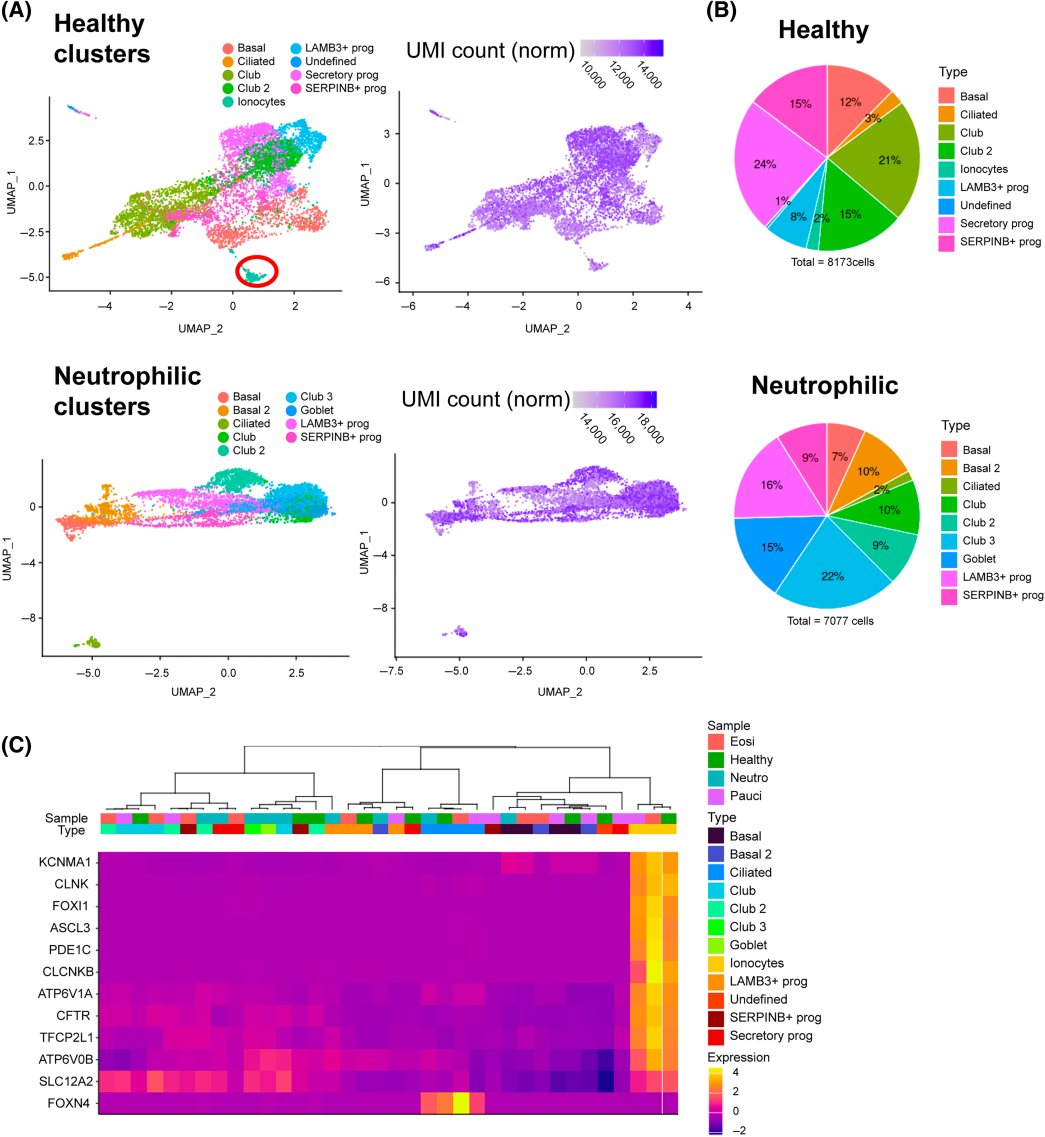
Single‐cell RNA‐seq analysis of hBECs. Single‐cell RNA‐seq was performed on bronchial epithelial cells at ALI generated from healthy subjects and neutrophilic asthma patients (*n* = 4 each). (A) Cells were clustered by using a graph‐based shared nearest neighbour method and plotted by UMAP together with heatmaps of gene UMI counts. Nine clusters of cells were identified in all samples and then known cell types were classified using classical gene markers enriched in the clusters through leadingEdge. Transitory progenitors or non‐classified cell types were identified by common transcriptional signatures SERPINB+ prog, LAMB3+ prog or Undefined. (B) The proportion of each cell subtype in healthy (8173 cells) and neutrophilic asthma (7077 cells) subjects are expressed (ionocytes cluster indicated in red circle has a proportion in healthy = 2%, ionocytes absent in neutrophilic asthma). (C) Unsupervised hierarchical clustering of all patient samples and cell subtypes (columns) by known ionocyte marker genes (rows), visualized using a heatmap plot.

We observed several minor abundance differences in classical subsets between healthy donors and severe asthma subtypes, however, there were two very clear differences revealed by the scRNAseq analysis. Bronchial epithelial cells from patients with neutrophilic asthma showed a marked increased of mature goblet cells compared to more immature secretory progenitors present in the other patient groupings (Figure 2A,B and Figure S1B,C in the Supporting Information). Furthermore, the most striking difference between patient samples was the complete loss of airway epithelial CFTR‐expressing ionocytes in patients with neutrophilic asthma (no ionocytes were detected among four independent patient samples of hBECs, 0% in over 8000 epithelial cells) (Figure 2A,B). Similarly, there was also a reduction of ionocyte abundance in paucigranulocytic samples compared to the higher abundance detected in hBECs from healthy donors (~2% of cells were ionocytes) and eosinophilic asthma (~2% of cells were ionocytes).

These differences were confirmed by unsupervised hierarchical clustering of ionocyte signature genes demonstrating the absence of these cells and their transcriptional marker genes in neutrophilic asthma (Figure 2C and Figure S2 in the Supporting Information). These data reveal a reduction of ionocyte differentiation in non‐eosinophilic asthma and provide a mechanism to explain the reduced CFTR and dysregulated ion channel function in differentiated hBECs from non‐eosinophilic asthma (Figure 1). Some other clusters shared features of multiple subtypes and were therefore likely in a progenitor or transitory stage, not a classical terminally differentiated airway epithelial subtype, and thus we labelled them by their predominant expression across patient groups of *SERPINB+* or *LAMB3+* as *SERPINB+* progenitors and *LAMB3+* progenitors. Secretory progenitors refers to cells expressing features of both club and goblet cells such as MUC5B but lacking most of the mature cell markers of either subset.

### Ionocytes from trachea or bronchi possess a conserved gene signature

**FIGURE 3:**
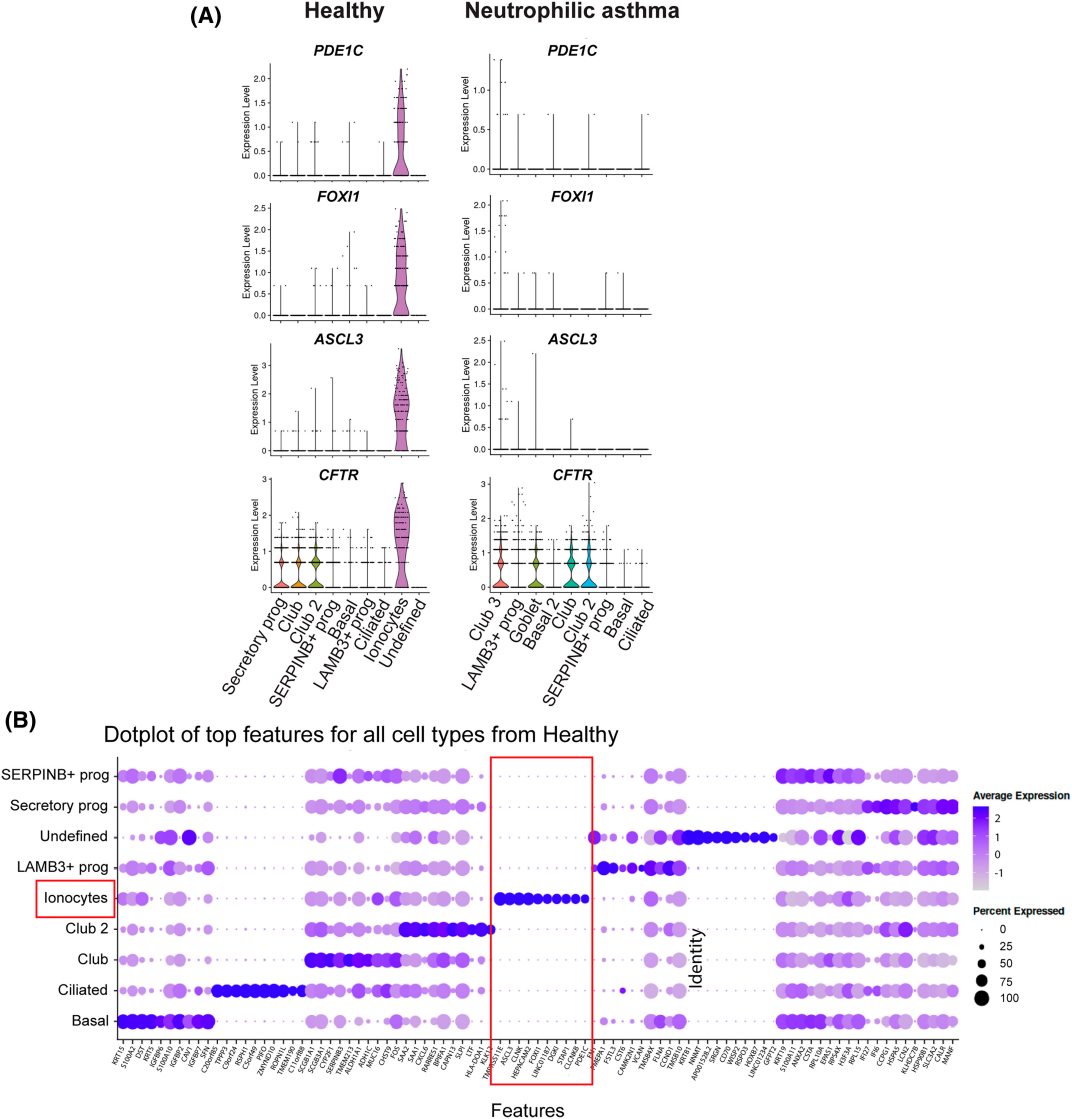
Identification of top ionocyte expressed genes in scRNAseq. (A) Violin plots of expression of ionocyte gene markers (*PDE1C, FOXI1, ASLC3* and *CFTR*) in each of the 9 cell subtype clusters from healthy subjects versus neutrophilic asthma patients. (B) Weighted dot plot showing top 10 features of each cluster from the scRNAseq data derived from cells of healthy subjects. Each dot is sized to represent the percent of cells in each cluster expressing the corresponding top 10 gene, and colours represent the average expression of each maker gene across within that cluster.

Expression of known ionocyte markers *FOXI1, ASCL3, PDE1C, TFCP2L1* and *CFTR* in single cells was lost in hBECs from patients with neutrophilic asthma (and reduced in paucigranulocytic asthma) compared to healthy donors or eosinophilic asthma (Figures 2, 3 and Figure S3 in the Supporting Information). This analysis also identified the top 10 most differentially expressed genes in ionocytes from hBECs (*TMPRSS11E, ASCL3, CLNK, HEPACAM2, FOXI1, LINC01187, DGKI, STAP1, CLCNKB* and *PDE1C*) compared to all other epithelial cell subset clusters through pairwise comparison with every other cell cluster (Figure 3B and Figure S4A–C in the Supporting Information). Similarly, this analysis identified classical transcriptional markers of other airway epithelial cell subsets. In neutrophilic asthma we observed increased secretory cells, in particular goblet cells and a subtype of club cells (club 3, similar to other club cells), club 3 top marker genes (Figure S4A in the Supporting Information) that distinguish them from other subtypes in addition to markers listed in Table S3 in the Supporting Information included *BPIFA1, IGFBP5, EGLN3, CXCL6, CSTB, CFD, PROM1* and *PNCK*. Goblet cell top marker genes (Figure S4A in the Supporting Information) that distinguish them from other subtypes in addition to known markers are listed in Table S3 in the Supporting Information and included *CD74, HLA, LCN2, FTH1, GDF15, NAMPT, AZGP1* and *EIF1AY*.

**FIGURE 4:**
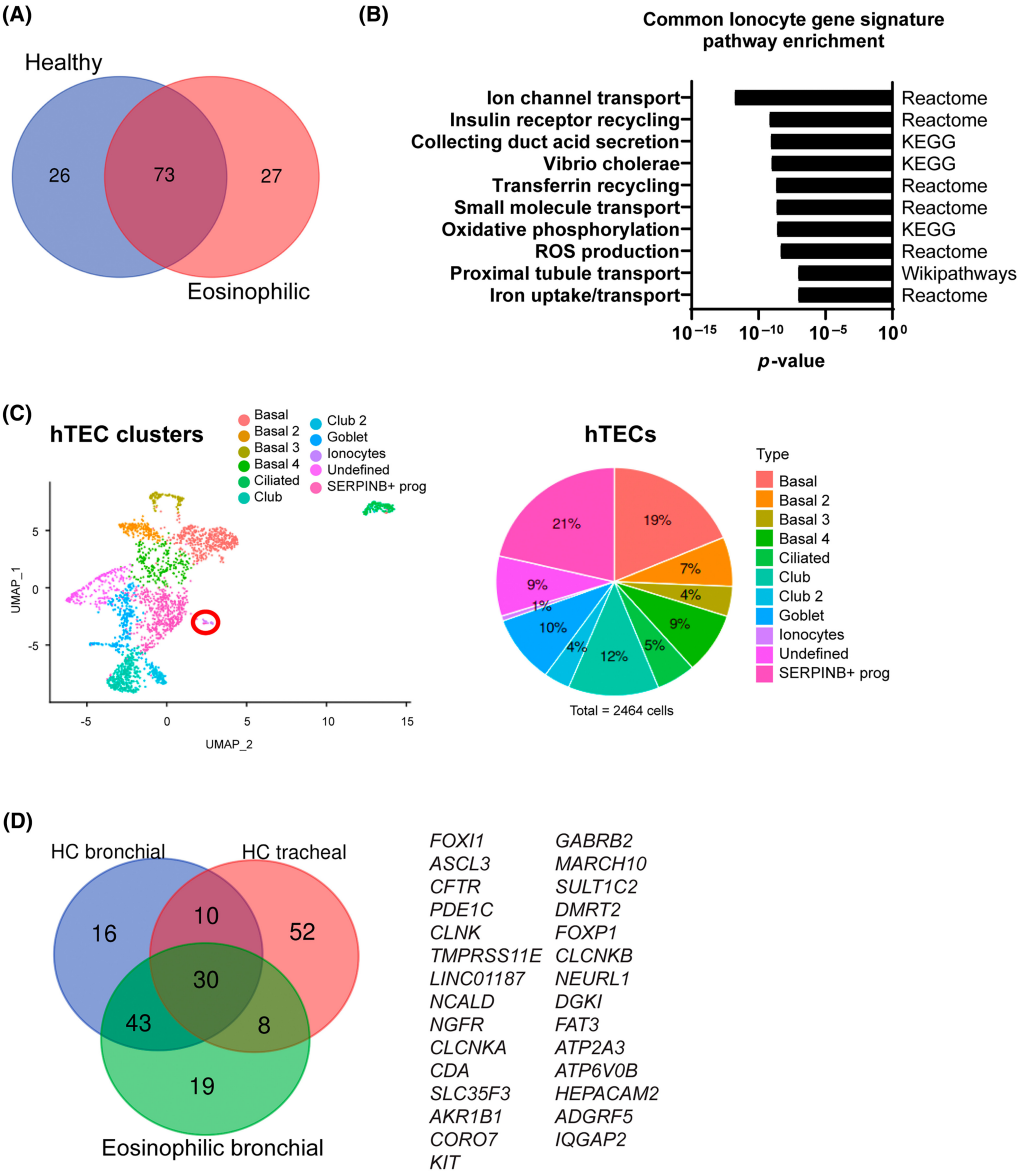
Pathway analysis of ionocyte gene signature and conservation throughout conducting airways. (A) Venn diagram of the 73 common ionocyte genes between hBECs from healthy subjects and eosinophilic asthma patients. (B) Pathway over‐representation analysis using ConsensusPathDB of the 73 common ionocyte gene signature. (C) Human tracheal epithelial cells (hTECs) cultured at ALI from healthy donors (*n* = 4), were clustered by using a graph‐based shared nearest neighbour method and plotted by UMAP. Eleven clusters of cells were identified and then known cell types were classified using classical gene markers enriched in the clusters through leadingEdge. Transitory progenitors or non‐classified cell types were identified by transcriptional signatures SERPINB+ prog, or as Undefined. The proportion of each cell type among hTECs was expressed in a pie chart (2464 cells). (D) Venn diagram of the conserved 30 common ionocyte gene signature among healthy hBECs, healthy hTECs and hBECs from eosinophilic asthma. HC, healthy control.

Through pairwise comparison in each sample for each cell cluster, the top 100 marker genes characteristic of ionocytes in both healthy donors and severe eosinophilic asthma were identified and these 100 genes had 73% overlap (Figure 4A). These genes were enriched in pathways for ion channel transport including chloride, sodium, iron and potassium ions, as well as pH regulation (Figure 4B and Figure S5A–C in the Supporting Information). Human phenotype ontology analysis linked ionocytes and their gene expression to physiologic salt imbalances and metabolic pH dysregulation (Figure S5C in the Supporting Information).

To determine if the ionocyte gene signature was conserved between different anatomical sites in the airways, we conducted scRNAseq on hTECs from healthy tissue also differentiated at ALI. Similar to bronchial cells we detected ionocytes in ~1% of hTECs (Figure 4C). We examined pathways over‐represented in the top 100 genes in the tracheal ionocyte gene signature and observed enrichment for ion channel activity and ion homeostasis as well as involvement of pathways in neuronal and immune signalling (Figure S6A–C in the Supporting Information).

To compare the similarity between the top gene signatures in samples with clear ionocyte populations, we examined common marker genes in each of the healthy hTEC and hBEC samples as well as hBECs from patients with eosinophilic asthma. We identified a conserved ionocyte signature of 30 marker genes among these samples derived from different anatomical sites and 12 different patients (30% common genes between the three sets of patient samples) (Figure 4D). This signature included the characteristic marker genes *FOXI1, ASCL3* and *CFTR* as well as multiple novel markers linked to neuronal signalling. Pathway analysis showed strong enrichment for genes involved in ion channel transport (Figure 4D and Figure S7A,B in the Supporting Information). All gene lists with the top 100 ionocyte markers genes for each category are in Table S4 in the Supporting Information.

### Quantification of ionocytes by protein markers confirms their loss in non‐eosinophilic asthma

**FIGURE 5:**
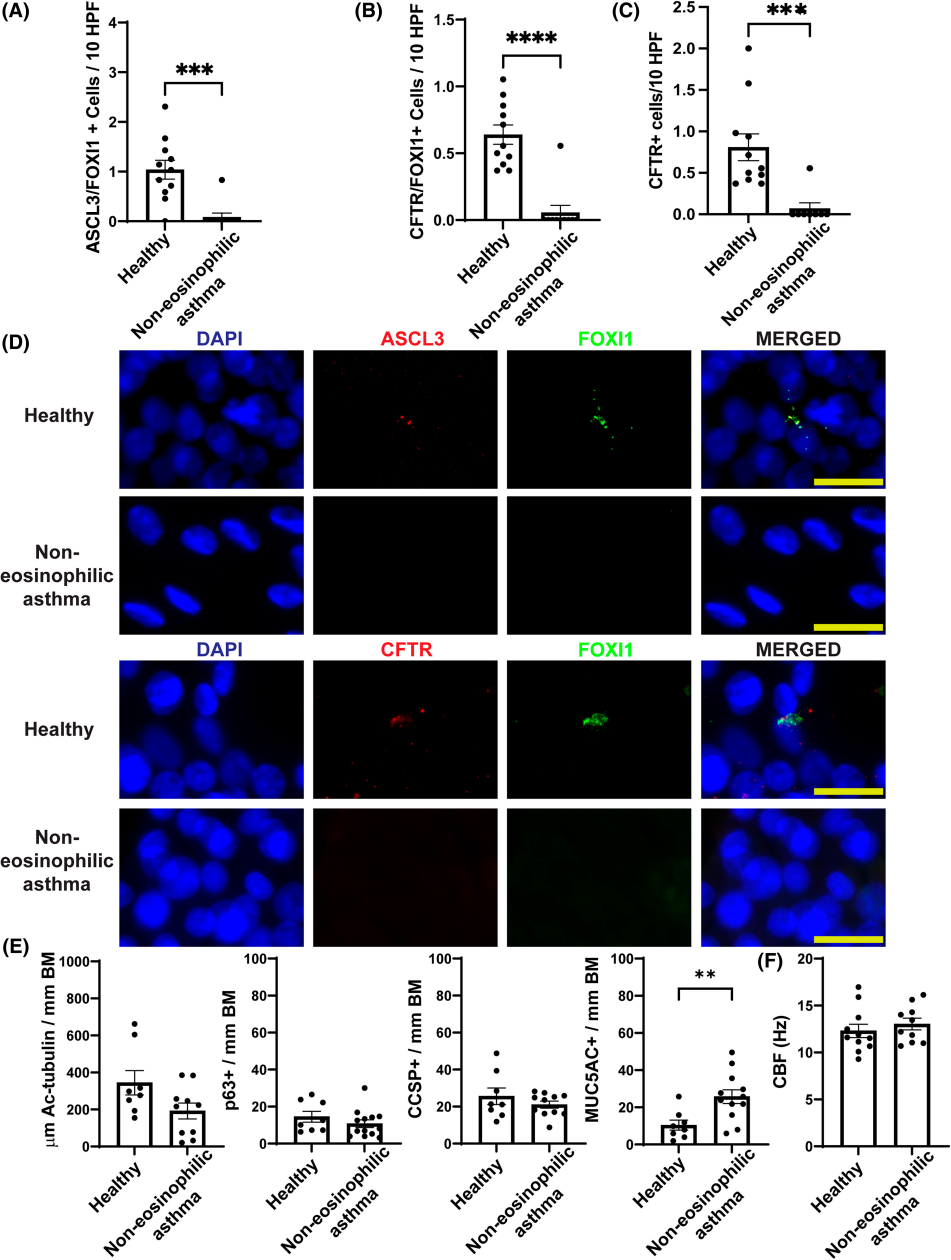
Quantification of ionocytes in hBECs by dual protein marker expression. Transwell membranes of ALI hBEC cultures from healthy or non‐eosinophilic asthma were used for dual marker immunofluorescent quantification of ionocytes and other airway epithelial subtypes. (A) Dual ASCL3 + FOXI1+ cells or (B) CFTR + FOXI1+ cells or (C) total CFTR+ cells were quantified by immunofluorescence in hBECs from healthy donors (*n* = 11) or non‐eosinophilic asthma (*n* = 10). Values expressed per 10 high‐powered fields (HPF), mean ± SEM. (D) Representative immunofluorescent images for (A) and (B), scale bars represent 20 μm. (E) Quantification of common airway epithelial cell subtypes; ciliated (Ac‐tubulin+), basal stem cells (p63+), club cells (CCSP+) and goblet (MUC5AC+) by immunofluorescence in hBECs from healthy donors (*n* = 8) or non‐eosinophilic asthma (*n* = 10–13). Values expressed per mm of basement membrane (BM), mean ± SEM. (F) Cilia beat frequency (CBF) measured in live images from hBECs of healthy (*n* = 11) versus non‐eosinophilic asthma (*n* = 10). Values expressed in Hz as mean ± SEM. Unpaired *t* test was used to determine statistical significance, **p < 0.01, ***p < 0.001 and ****p < 0.0001.

Using another cohort of patient hBECs differentiated at ALI we sought to validate the loss of ionocytes in non‐eosinophilic asthma that we observed through scRNAseq. We quantified the ionocyte markers FOXI1, ASCL3 and CFTR by dual immunofluorescent protein quantification, which confirmed a significant (almost complete) loss of ionocytes (measured by dual labelling ASCL3 + FOXI1+ and CFTR + FOXI1+) in hBECs of non‐eosinophilic asthma (Figure 5A–D). Although *CFTR* is expressed at a lower level in other airway epithelial cell subtypes, in particular secretory cells, the highest expression of *CFTR* was in ionocytes from both healthy and asthma donors (Figure 3A), which also validated the reduced but remaining residual CFTR function in hBECs from patients with non‐eosinophilic asthma (Figure 1A). At the protein level we confirmed this reduction in CFTR expressing cells in non‐eosinophilic asthma (Figure 5C), noting that protein‐antibody immunofluorescent staining is known to be less sensitive for CFTR than detection of RNA or CFTR function by ion current, and so is more likely to detect cells with a higher abundance of CFTR. We examined the impact of increasing secretory cell differentiation in the hBECs via recombinant IL‐13 treatment on the total number of CFTR protein‐expressing cells. As expected, this cytokine treatment increased secretory cells in the cultures, but it did not significantly increase CFTR expression (Figure S8A–C in the Supporting Information).

Similar to our scRNAseq results, the immunofluorescent protein quantification showed there was a significant reduction of ionocytes in paucigranulocytic asthma hBECs and a complete loss in neutrophilic asthma (Figure S8D,E in the Supporting Information). By examining the relative *CFTR* distribution on different airway epithelial cell types we observed that in healthy donors ionocytes were the primary cells expressing *CFTR* but with clear expression of transcript in club cells and the secretory/goblet lineage (Figure S8F in the Supporting Information). In neutrophilic asthma ionocyte expression was lost but residual *CFTR* transcript remained present in club subtypes as well as the secretory/goblet lineage (Figure S8G in the Supporting Information). This *CFTR* transcript level in the non‐ionocyte cell types remained relatively stable between healthy donors and neutrophilic asthma (Figure S8H in the Supporting Information).

Established protein markers of other airway epithelial cell subsets were also quantified and showed no significant changes between healthy and non‐eosinophilic asthma hBECs including; acetylated tubulin+ ciliated cells, p63+ basal stem cells or CCSP+ club cells, nor was there any functional difference in ciliated cell beat frequency between the patient hBECs (Figure 5E,F and Figure S9A,B in the Supporting Information).

Quantification of MUC5AC+ cells showed a significant increase in mature goblet cells in non‐eosinophilic asthma (reflecting our scRNAseq results) (Figures 2 and 5D). Furthermore, these patients show increased clinical symptoms of mucus over‐production (CAT1 + CAT2 scores, Table S1 in the Supporting Information). Goblet cell hyperplasia and mucus hypersecretion are also features of other chronic airways disease with a dysfunctional CFTR pathway including CF and COPD.^24^

These results validated the findings from the scRNAseq discovery set analysis. Overall, these data clearly demonstrate a loss of ionocytes and increase in mature goblet cells in hBECs from non‐eosinophilic asthma. Ionocytes through their high expression of CFTR are established to regulate CFTR anion transport, therefore we assessed whether there was a correlation between ionocyte numbers in patients with severe non‐eosinophilic asthma plus controls compared to CFTR ion currents. We measured this using two different immunofluorescent staining approaches and found that both ASCL3 + FOXI1+ as well as CFTR + FOXI1+ ionocyte numbers were significantly correlated with changes in CFTR ion transport (Pearson *r* = 0.55 or 0.66, respectively) (Figure S9C,D in the Supporting Information).

### Loss of ionocytes is mediated via neutrophilic asthma‐associated cytokines, in particular IFN‐γ

**FIGURE 6:**
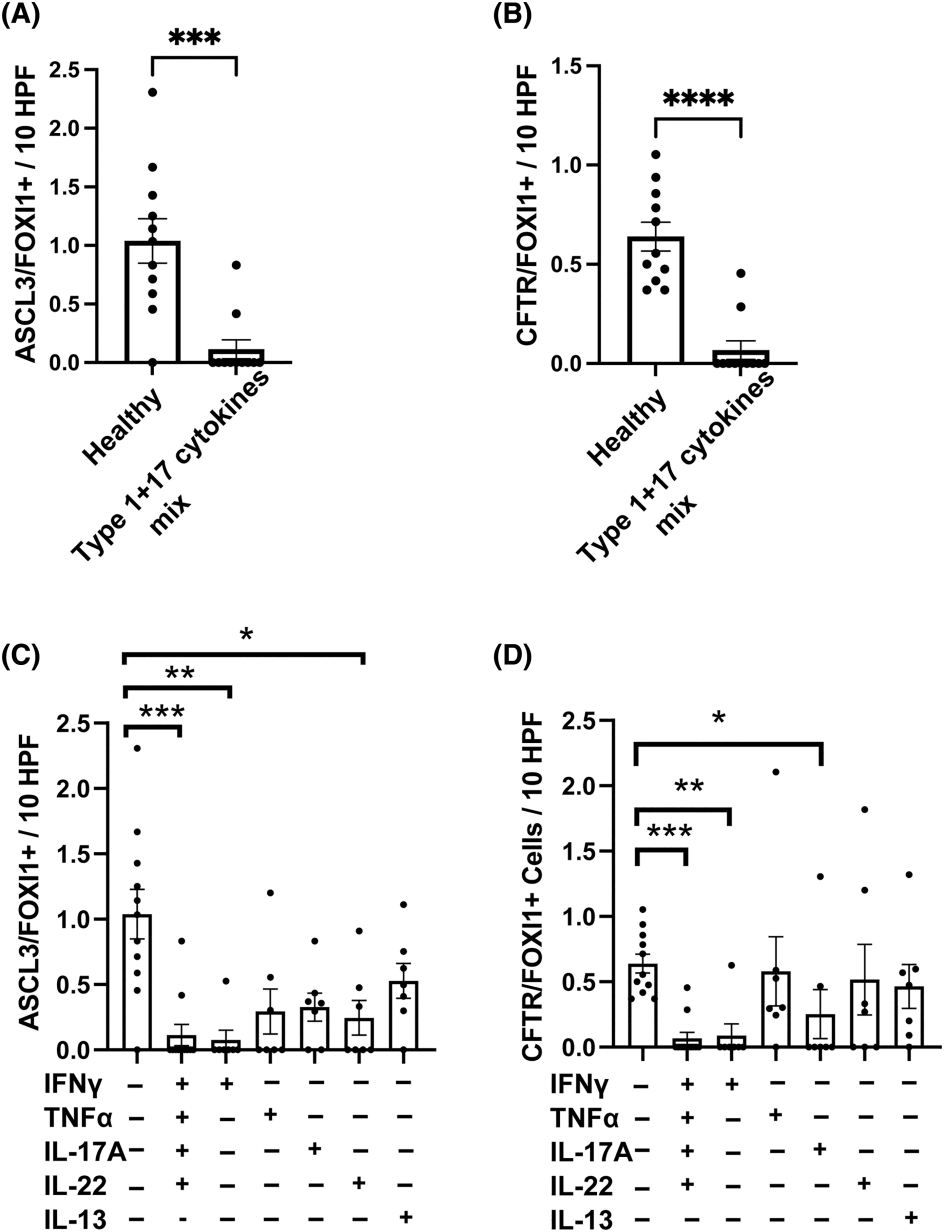
Inflammatory cytokine‐induced loss of ionocytes. Transwell membranes of ALI hBEC cultures from healthy donors were used for dual marker immunofluorescent quantification of ionocytes. (A and C) Dual ASCL3 + FOXI1+ cells or (B and D) CFTR + FOXI1+ cells were quantified by immunofluorescence in healthy hBECs treated with vehicle control, a cytokine mixture representative of non‐eosinophilic asthma airway secretions including IFN‐γ, IL‐17A, IL‐22 and TNF‐α (*n* = 11 each), or (C and D) with the individual cytokines indicated (*n* = 7 each). Values expressed per 10 high‐powered fields (HPF), mean ± SEM. Paired *t* test (A, B) and Kruskal–Wallis with Dunn's multiple comparison test (C, D) were used to determine statistical significance. *p < 0.05, ***p < 0.001 and ****p < 0.0001.

We hypothesized that the loss of CFTR‐expressing ionocytes in non‐eosinophilic asthma is likely an acquired phenotype, mediated by inflammation present in severe non‐eosinophilic asthma and in particular neutrophilic asthma. The latter is characterized by elevated expression of Type 1 and Type 17 cytokines including IFN‐γ, IL‐17A, TNF‐α and IL‐22 in patient airway tissue biopsies, sputum and BAL.^3^, ^15^, ^25^, ^26^ To assess if these cytokines associated with neutrophilic asthma might lead to inflammation‐induced loss of ionocytes, hBECs from healthy donors at ALI (which contain ionocytes) were treated with individual cytokines or a representative neutrophilic asthma mixture (Type1 + 17 cytokine mix: IFN‐γ, IL‐17A, TNF‐α and IL‐22). Immunofluorescent quantification by both ASCL3 + FOXI1+ and CFTR + FOXI1+ both showed a dramatic reduction in ionocytes after treatment with the cytokine mixture (Figure 6A,B and Figure S10A,B in the Supporting Information).

Treatment of these hBECs with individual cytokines showed this effect was primarily due to IFN‐γ, with a much smaller and only partial effect of the Type 17 cytokines, IL‐17A and IL‐22 (Figure 6C,D). In contrast, there was no significant impact of the Type 2 cytokine IL‐13 on ionocyte abundance by either measure. Similarly, asthmatics with high airway eosinophils and elevated Type 2 cytokine linked FeNO have a clear population of ionocytes (Table S1 and Figure S1 in the Supporting Information) unlike non‐eosinophilic asthma with higher neutrophils and lower FeNO. These data demonstrate that some specific inflammatory cytokines characteristically elevated in neutrophilic asthma airways can reduce the abundance of ionocytes.

### Loss of ionocytes in a murine model of neutrophilic asthma, but not eosinophilic asthma

Using LINCS L1000 Ligand Perturbations database and inputting our ionocyte gene signature this in silico prediction database suggested that IFN‐γ was the most highly predicted molecule to regulate our ionocyte gene signature, which also complemented the experimental findings from our human in vitro results (Figure S11A in the Supporting Information). In addition, given the known involvement of the NOTCH pathway with FOXI1 and ionocyte development^27^ we assessed whether IFN‐γ may impact NOTCH pathway activation. HES1 is directly activated downstream of NOTCH and a commonly used readout for NOTCH pathway signalling. Our data showed that treatment with IFN‐γ reduced the expression of the *HES1* gene transcript by ~4‐fold (Figure S11B in the Supporting Information). To determine if inflammation‐induced loss of ionocytes also occurs in vivo, we quantified abundance of ionocytes in established murine models of eosinophilic/Type 2 high asthma versus neutrophilic/Type 2 low asthma.

We compared asthma models that are induced by the antigen ovalbumin (OVA) or the allergenic fungus *Alternaria alternata* (AA), which are Type 2 high disease models characterized by airway eosinophilia, versus a neutrophilic asthma model using the intracellular respiratory bacteria *Chlamydia muridarum* plus ovalbumin (Cmu/OVA), which produces neutrophil‐dominant airway inflammation (Figure S12A in the Supporting Information). These well‐established models are driven by IL‐4/‐5/‐13 or IFN‐γ/IL‐17A, respectively.^18^, ^19^, ^28^, ^29^ We quantified ionocyte numbers in bronchial tissue measured by dual immunofluorescent quantification by both ASCL3 + FOXI1+ and CFTR + FOXI1+ (Figure S12B–E in the Supporting Information). Ionocytes were significantly diminished in the Cmu/OVA neutrophilic asthma model, whereas these cells remained readily detectable in the eosinophilic models with either AA or OVA as well as controls (Figure S12D,E in the Supporting Information). These in vivo data clearly support our in vitro studies with human airway epithelial cells demonstrating that neutrophilic asthma associated inflammation can induce loss of CFTR‐expressing ionocytes.

Finally, we sought to determine whether CFTR function could be improved in neutrophilic asthma hBECs using the existing standard‐of‐care CFTR modulator therapy Elexacaftor/tezacaftor/ivacaftor (VX445/VX661/VX770) that is used in the treatment of cystic fibrosis to improve CFTR protein folding and trafficking at the membrane. Our results demonstrated that this drug combination significantly improved CFTR function in airway epithelial cells from patients with CF, however, it failed to do so in those derived from patients with neutrophilic asthma or healthy donors (Figure S12F in the Supporting Information). This result is somewhat expected given the CFTR functional deficiency we have identified in neutrophilic asthma is due to a reduction in the number of CFTR‐expressing ionocytes, rather than a CFTR protein‐folding, trafficking or gating defect, which is the problem in the CF genotypes treated by modulators.

## DISCUSSION

Insights into the pathogenesis of non‐eosinophilic and neutrophilic asthma may be gained by examining the overlap with cellular and pathological markers of other chronic airways diseases including COPD, CF and subsets of non‐CF bronchiectasis. These diseases also show strong neutrophilic infiltrates in the airways, a dominant mixture of Type 1 and Type 17 cytokine responses, mucus hypersecretion and high airway bacterial colonization.^5^, ^6^, ^7^, ^8^, ^9^, ^10^, ^30^ Indeed, the characteristic airway colonizers of CF, *Pseudomonas aeruginosa* and *Haemophilus influenzae*, are abundant in COPD and non‐CF bronchiectasis and are also markers of the non‐eosinophilic asthma microbiome.^31^, ^32^, ^33^, ^34^, ^35^ These similarities raise the possibility of common mechanistic drivers. Defective CFTR is sufficient to drive these disease features in CF, and is linked to this microbial phenotype in a proportion of non‐CF bronchiectasis cases.^36^ Smoking is known to reduce the function of CFTR, resulting in acquired loss of CFTR activity in COPD.^37^, ^38^ A small case series report has also linked non‐allergic asthma to abnormal CFTR function observed through nasal potential differences,^11^ providing a clue that defects in this pathway could be linked to the clinical phenotype of non‐eosinophilic asthma through a feedback mechanism.

Although there is evidence, some of it conflicting, that the risk of asthma is higher in non‐CF individuals with *CFTR* gene variants (i.e., *CFTR* carrier heterozygosity), the data suggests that genetic variants are unlikely to be a dominant causal factor in non‐eosinophilic asthma.^39^, ^40^ Therefore, any defects in CFTR activity in non‐eosinophilic asthma are likely acquired or induced rather than genetic.

Based on this rationale we investigated CFTR function in airway epithelial cells from patients with non‐eosinophilic asthma and observed a substantial reduction (~50% loss) in CFTR function and protein expression. This confirmed our hypothesis that the CFTR pathway is dysfunctional in airway epithelial cells from severe non‐eosinophilic asthma. To provide greater mechanistic depth to this observation we used scRNAseq to analyse hBECs from severe asthma subtypes and healthy controls. scRNAseq detects transcriptional changes at the individual cell level and rarer cell types can be studied that would be missed with the more traditional transcriptional approaches of bulk RNAseq, microarrays or targeted PCR. We discovered through scRNAseq that the CFTR‐rich cell type, the airway ionocyte, was diminished in hBECs from non‐eosinophilic asthma. More specifically this cell type was almost completely lost in neutrophilic asthma and significantly diminished in paucigranulocytic asthma, which we confirmed using immunofluorescent dual labelling and quantification of known ionocyte markers.

Both scRNAseq and confirmatory protein cell marker validation showed that other epithelial subtypes remain largely unchanged apart from an increase in mature goblet cells in neutrophilic asthma. Furthermore, the CAT1 plus CAT2 score for mucous production and chronic bronchitis was significantly higher in the neutrophilic asthma patients, suggesting that clinically these patients were also experiencing higher mucus production and cough than eosinophilic asthma patients (Table S1 in the Supporting Information). As goblet cell hyperplasia and mucus hypersecretion are observed in other chronic airways diseases linked to CFTR dysfunction (CF and COPD) this provided further evidence of a role for CFTR in non‐eosinophilic asthma.

Using the cell cluster marker genes we identified in ionocytes in hBECs from healthy controls and eosinophilic asthma, as well as those identified in healthy hTECs, we discovered a conserved ionocyte gene signature present in epithelial cells from the trachea to the third‐fourth branch of the bronchi. This was a markedly conserved gene signature (~30%) from three different donor groups (healthy control hBECs or hTECs, and eosinophilic asthma hBECs). This signature confirmed previously known ionocyte markers^27^, ^41^ such as the transcription factor *FOXI1*, *ACSL3*, *CFTR*, *PDE1C* and *ATP6V0B* but also identified new markers novel to ionocyte function including *NEURL1*, a Notch pathway regulator, and *NCALD*, *GABRB2* and *NGFR* all involved in neural signalling and neurotransmission.

Future studies will be required to investigate the interaction of ionocytes and neural signalling processes in the airways. Overall, pathway analysis clearly showed that the ionocyte gene signature was linked to ion channel transport for chloride, iron, and potassium and pH regulation. This would suggest these cells could be important in a range of chronic airways diseases where ion homeostasis and pH are dysregulated. Indeed, asthma patients with lower lung function and more severe symptoms have lower airway pH detected in exhaled breath condensate and dysregulated iron homeostasis.^42^, ^43^

Our scRNAseq analysis of hBECs also identified two subpopulations of clusters of epithelial cells (we referred to as *LAMB3+* or *SERPINB+* progenitors) without a clear delineated differentiated cell identity and thus are likely transitioning between subtypes. These clusters expressed a limited number of markers in common with basal cells, such as *SOX* gene family members, cyclin cell cycle genes and in the case of one of the populations the basal cell gene *LAMB3*, suggesting they may have progenitor potential. These subpopulation percentages were similar across all patient groups. These clusters likely represent a transitory or plastic stage of differentiation of the airway epithelial cells. The *LAMB3+* progenitor cluster had a signature enriched for some epithelial‐mesenchymal genes and the *SERPINB+* progenitor cluster had genes involved in protease/anti‐protease pathways. Future studies will need to confirm these subpopulations in vitro and in vivo and investigate their cellular function.

Our data demonstrated that ionocytes are lost in hBEC ALI cultures from patients with non‐eosinophilic asthma, and we hypothesized that this is likely an acquired or induced defect caused by inflammatory pathways. Differentiated ALI cultures of airway epithelial cells retain functional and morphological characteristics present in human diseases like asthma and COPD in vivo such as p63+ basal stem cell hyper‐proliferation, diminished interferon and anti‐viral responses and increased goblet cells.^24^, ^44^, ^45^, ^46^, ^47^ ALI airway epithelial in vitro cultures also reflect the transcriptional activity of fresh ex vivo airway epithelial brushings.^48^, ^49^ Thus, we postulated an inflammation‐induced loss of ionocytes and CFTR function was responsible for the reduction observed in hBECs from patients with non‐eosinophilic asthma. This would constitute an ‘inflammatory memory’ as proposed by *Ordovas‐Montanes* et al., being programmed into the epithelial progenitor cells.^50^

The airways of patients with non‐eosinophilic asthma, in particular the neutrophilic subset, are consistently found to be dominated by Th1 and Th17 cytokines in BAL, endobronchial biopsies and sputum with elevated levels of IL‐17A, IL‐22, IFN‐γ and TNF‐α.^3^, ^15^, ^25^ Based on this knowledge we treated hBECs from healthy non‐asthmatic donors with these cytokines in a cocktail, or individually, and demonstrated that these mediators of neutrophilic airway inflammation are sufficient to dramatically reduce the numbers of CFTR‐expressing ionocytes. In particular, the Type 1 cytokine IFN‐γ, whose protein levels and downstream gene signatures are enriched in neutrophilic asthma^3^, ^15^, ^25^ almost completely inhibited ionocyte differentiation. Previous work has shown that the combination treatment of TNF‐α and IL‐17A for 24 h led to a small increase in CFTR expression (and a much more substantial increase in the expression of the Pendrin HCO^3−^ transporter).^51^ Our results are not directly comparable to this work as we did not co‐treat with only these cytokines, and we tried to mimic chronic neutrophilic airway inflammation in asthma by treating hBECs over 2 weeks with cytokines, however, our results suggest a partial loss of CFTR‐expressing ionocytes with IL‐17A under these conditions.

Although we observed a consistent loss of ionocytes in neutrophilic asthma and these cells are high expressors of CFTR, we still observed a clear residual CFTR‐dependent current, which was matched and explained by the clear presence of *CFTR* RNA transcript in other epithelial cell subtypes, primarily club and goblet/secretory progenitor cell populations. However, this residual CFTR was more difficult to detect at the protein level with CFTR antibodies, which is likely due to the reduced sensitivity of CFTR antibodies in general and their ability to mainly detect high CFTR abundance cells more readily than low CFTR abundant cells.

To confirm these in vitro results using an in vivo approach we used multiple mouse models of asthma‐like airway pathology that recapitulate either a classical Type 2 cytokine/eosinophilic‐dominant airways disease (through sensitisation and challenge with ovalbumin [OVA] or *Alternaria alternata* [AA]), or a more neutrophilic‐dominant airways disease characterized by elevated Th1 and Th17 cytokines induced by *Chlamydia muridarum* infection during OVA challenge.^18^, ^19^, ^28^, ^29^ Our in vivo findings from these models, similar to our in vitro data, showed an almost complete loss of airway ionocytes in the presence of neutrophilic airway disease, but not eosinophilic‐dominant disease. These studies add further weight to our observations that inflammation‐induced loss of ionocytes and CFTR is a feature of non‐eosinophilic asthma. Future studies will be necessary to determine whether ionocytes have a role in mediating airway ion transport homeostasis and regulating airway pH, other than through the CFTR, and how this impacts asthma pathogenesis in these models.

We showed that the best‐in‐class CFTR modulator Elexacaftor/tezacaftor/ivacaftor is unable to rescue CFTR function in neutrophilic asthma hBECs, because this drug combination works by improving folding, trafficking and membrane openness of a mutated CFTR ion channel in CF, but it is not efficacious in CF patients where there is a lack of CFTR expression. Therefore, given we have identified the abnormality in neutrophilic asthma to be a lack of CFTR‐expressing ionocytes it is not surprising that this drug does not work to reverse this. Future therapies that either target boosting of CFTR gene expression, or work to reduce the inflammatory milieu that suppresses it, are likely needed to improve this pathologic pathway in patients with neutrophilic asthma.

Limitations of this work that should be addressed in future studies include the validation of these phenotypes in fresh epithelial brushings without culture, as well as how the levels of ionocytes and other airway epithelial subtype gene programs change in patients over time through longitudinal sampling, and whether this remains stable or not within severe asthma endotypes. Future investigations could also assess whether culture with the other major airway epithelial in vitro differentiation system using Pneumacult impacts the presence of airway cell subtypes derived from asthma patients, as multiple reports have indicated that the choice between BEGM and Pneumacult can impact rarer cell numbers including ionocytes.^52^, ^53^ However, it is important to note that these studies use conditionally reprogrammed cell expansion, which can cause the loss of some patient cell phenotypes in vitro, hence differences in ionocyte numbers between these differentiation media types may not occur with freshly isolated and cultured hBECs.

This work highlights that an important pathogenic ion transport pathway through the CFTR and ionocytes are dysregulated in severe non‐eosinophilic asthma. Given there is an established role of the CFTR pathway in other chronic airway diseases, our findings describing the loss of CFTR‐rich ionocytes may encourage further studies to evaluate CFTR regulating therapies for difficult‐to‐treat non‐eosinophilic asthma.

## AUTHOR CONTRIBUTIONS

**Ling Chen:** Formal analysis (equal); investigation (equal); methodology (equal); writing – review and editing (supporting). **Gabriela Araujo Hoefel:** Formal analysis (equal); investigation (equal); methodology (equal); writing – review and editing (supporting). **Prabuddha S. Pathinayake:** Investigation (supporting); methodology (supporting); writing – review and editing (supporting). **Andrew Reid:** Investigation (supporting); writing – review and editing (supporting). **Amber L. Pillar:** Investigation (supporting); writing – review and editing (supporting). **Coady Kelly:** Investigation (supporting); writing – review and editing (supporting). **Tan HuiYing:** Methodology (supporting); writing – review and editing (supporting). **Ayesha Ali:** Investigation (supporting); methodology (supporting). **Richard Y. Kim:** Resources (supporting); writing – original draft (supporting). **Philip M. Hansbro:** Resources (supporting); writing – review and editing (supporting). **Steven L. Brody:** Resources (supporting); writing – review and editing (supporting). **Paul S. Foster:** Funding acquisition (supporting); resources (supporting); writing – review and editing (supporting). **Jay C. Horvat:** Investigation (supporting); resources (supporting); writing – review and editing (supporting). **Carlos Riveros:** Formal analysis (supporting); investigation (supporting); methodology (supporting); writing – review and editing (supporting). **Nikhil Awatade:** Formal analysis (supporting); investigation (supporting); validation (lead); writing – review and editing (supporting). **Peter A. B. Wark:** Conceptualization (equal); funding acquisition (supporting); resources (lead); writing – original draft (equal). **Gerard E. Kaiko:** Conceptualization (equal); funding acquisition (lead); project administration (lead); resources (supporting); writing – review and editing (equal).

## CONFLICT OF INTEREST STATEMENT

G. E. K. and L. C. have performed university contract research for Krystal Biotech on CFTR in cystic fibrosis but not related to this work, and not involved in the funding of this work. P. A. B. W. has received honoraria from GSK, Astra Zeneca, Sanofi, Novartis and Vertex but not for this work. P. A. B. W. is a committee member of the National Asthma Council of Australia. The other authors declare no conflicts of interest.

## HUMAN AND ANIMAL ETHICS APPROVAL DECLARATION

Approval of this study was obtained from Hunter New England Health human research ethics committee (05/08/10/3.09). Written informed consent was obtained from all study subjects. Mouse studies were performed in accordance with protocols for the care and use of animals approved by the Animal Ethics Committee of The University of Newcastle (A‐2009‐127 and A‐2017‐721).

## Supporting information


**Figure S1.** Characterization of patients with severe asthma subtypes and single‐cell RNA‐seq analysis. (A) Percentage from differential cell counts in BAL fluid from healthy subjects and asthma patients subtyped. Single‐cell RNA‐seq was performed on single‐cell suspensions generated from hBECs of eosinophilic asthma patients and paucigranulocytic asthma patients (*n* = 4 each). (B) Cells were clustered by using a graph‐based shared nearest neighbour method and plotted by UMAP together with heatmaps of gene UMI counts. Nine clusters of cells were identified in all samples and then known cell types were classified using classical gene markers enriched in the clusters through leadingEdge. Transitory progenitors or non‐classified cell types were labelled by predominant transcriptional signatures SERPINB+ prog, LAMB3+ prog or Undefined. (C) The proportion of each cell type in eosinophilic (9624 cells) and paucigranulocytic asthma (6914 cells) patients were calculated using pie charts.

**Figure S2.** Unsupervised hierarchical clustering of all patient samples and cell subtypes (columns) by known ionocyte and basal cell marker genes (rows), visualized using a heatmap plot.

**Figure S3.** (A) Violin plots of expression of ionocyte gene markers (*PDE1C, FOXI1, ASLC3* and *CFTR*) in each of the 9 cell subtype clusters from eosinophilic versus paucigranulocytic asthma patients.

**Figure S4.** Weighted dot plot showing top 10 features of each cluster from the scRNAseq data derived from cells of (A) neutrophilic, (B) eosinophilic and (C) paucigranulocytic asthma patients. Each dot is sized to represent the percent of cells in each cluster expressing the corresponding top 10 gene, and colours represent the average expression of each maker gene across within that cluster.

**Figure S5.** Pathway over‐representation analysis. (A) Gene Ontology (GO) analysis for Biological Process and (B) Molecular Process of the 73 common bronchial ionocyte genes. (C) Human Phenotype Ontology analysis of the 73 common bronchial ionocyte genes.

**Figure S6.** Pathway over‐representation analysis. (A) Gene Ontology (GO) analysis for Biological Process and (B) Molecular Process of the top 100 tracheal ionocyte genes. (C) Human Phenotype Ontology analysis of the top 100 tracheal ionocyte genes.

**Figure S7.** (A) Weighted dot plot showing top 10 features of each cluster from the scRNAseq data derived from human tracheal epithelial cells (hTECs) from healthy donors. Each dot is sized to represent the percent of cells in each cluster expressing the corresponding top 10 genes, and colours represent the average expression of each maker gene across within that cluster. (B) Pathway over‐representation analysis using ConsensusPathDB of the 30 common genes in the ionocyte gene signature from Figure 4D conserved between hBECs of healthy and eosinophilic asthma donors as well as hTECs.

**Figure S8.** Loss of ionocytes in non‐eosinophilic (neutrophilic and paucigranulocytic) asthma hBECs. (A) *CFTR* gene expression and (B) quantification of the number of CFTR protein expressing (CFTR+) cells in healthy hBECs treated with or without IL‐13 (*n* = 7–9). (C) Representative immunofluorescent images of healthy hBECs treated with or without IL‐13 showing increased numbers of CCSP+ and MUC5AC+ goblet cells. (D) ASCL3 + FOXI1+ and (E) CFTR + FOXI1+ in hBECs from neutrophilic (*n* = 6) and paucigranulocytic (*n* = 4) asthma or healthy donors (*n* = 11), values expressed as mean ± SEM. (F‐G) Pie chart relative distribution of CFTR transcript level in each cell type scaled by cluster size for healthy donors (F) or neutrophilic asthma (G). (H) CFTR transcript level in non‐ioncytes normalized by library size across samples as CFTR counts per million total reads. *p ≤ 0.05, **p < 0.01 and ****p < 0.0001.

**Figure S9.** (A, B). Representative immunofluorescent images of healthy (A) or non‐eosinophilic asthma hBECs (B) indicating cells positive for p63 (green), MUC5AC (green), Ac‐tubulin, p63 (green) or CCSP (red), scale bar is 20 μm. (C and D) Linear regression analysis with Pearson r correlation between changes in CFTR ion currents (Ussing chamber) and number of ionocytes per HPF (immunofluorescence).

**Figure S10.** Representative immunofluorescent images of healthy hBECs treated with or without Type 1 + 17 cytokine (IFN‐γ, IL‐17A, TNF‐α and IL‐22) mix showing (A) ASCL3 + FOXI1+ and (B) CFTR + FOXI1+ in hBECs from healthy donors.

**Figure S11.** (A) Library of Integrated Network‐Based Cellular Signatures (LINCS) L1000 ligand perturbations analysis showing cytokines and ligands most highly predicted to regulate the ionocyte gene signature. (B) *HES1* gene expression measured by qPCR normalized to *GAPDH* house‐keeper in healthy control hBECs treated with or without IFN‐γ.

**Figure S12.** Loss of ionocytes in vivo in a murine model of neutrophilic asthma and impact of CFTR modulators on CFTR function in neutrophilic asthma hBECs. (A) Number of neutrophils, eosinophils, lymphocytes, macrophages and total number of cells/mL in bronchoalveolar lavage (BAL) fluid in control, fungal allergen *Alternaria alternata* (AA) and protein antigen ovalbumin (OVA) models of T2 asthma, as well as the *Chlamydia muridarum* (Cmu)/OVA‐treated model of non‐eosinophilic asthma. (B and C) Representative immunofluorescent images of PBS control mouse airways stained with (B) Ascl3 plus Foxi1 or (C) Cftr plus Foxi1, scale bar is 20 μm. (D) Dual Ascl3 + Foxi1+ cells or (E) Cftr + Foxi1+ cells were quantified by immunofluorescence in the bronchi of mice from these asthma models (*n* = 7–11 mice per group). Values expressed as per airway, mean ± SEM. Kruskal–Wallis test with Dunn's multiple comparison test compared to control mice, *p < 0.05, ***p < 0.001 and ****p < 0.0001. (F) Airway epithelial cells cultured at ALI derived from healthy donors (*n* = 5), neutrophilic asthma (*n* = 5) or patients with cystic fibrosis genotype (homozygous F508del/F508del *n* = 6), were treated with control or the CFTR modulator combination VX445/VX661/VX770, data shows the delta values of short circuit currents (∆Isc) for CFTR function Forskolin + 3‐isobutyl‐1‐methylxanthine (IBMX) stimulated. Values expressed as per airway, mean ± SEM. Wilcoxon paired *t* test *p < 0.05.

**Data S1:** Supporting Information.

## Data Availability

De‐identified single cell RNA‐sequencing datasets are available upon request from the corresponding author.
