# Supplementary figures and images for: Inflammation‐induced loss of CFTR‐expressing airway ionocytes in non‐eosinophilic asthma
Source: Respirology. 2024 Oct 2;30(1):25–40. doi: 10.1111/resp.14833 (PMC11688627; doi:10.1111/resp.14833)

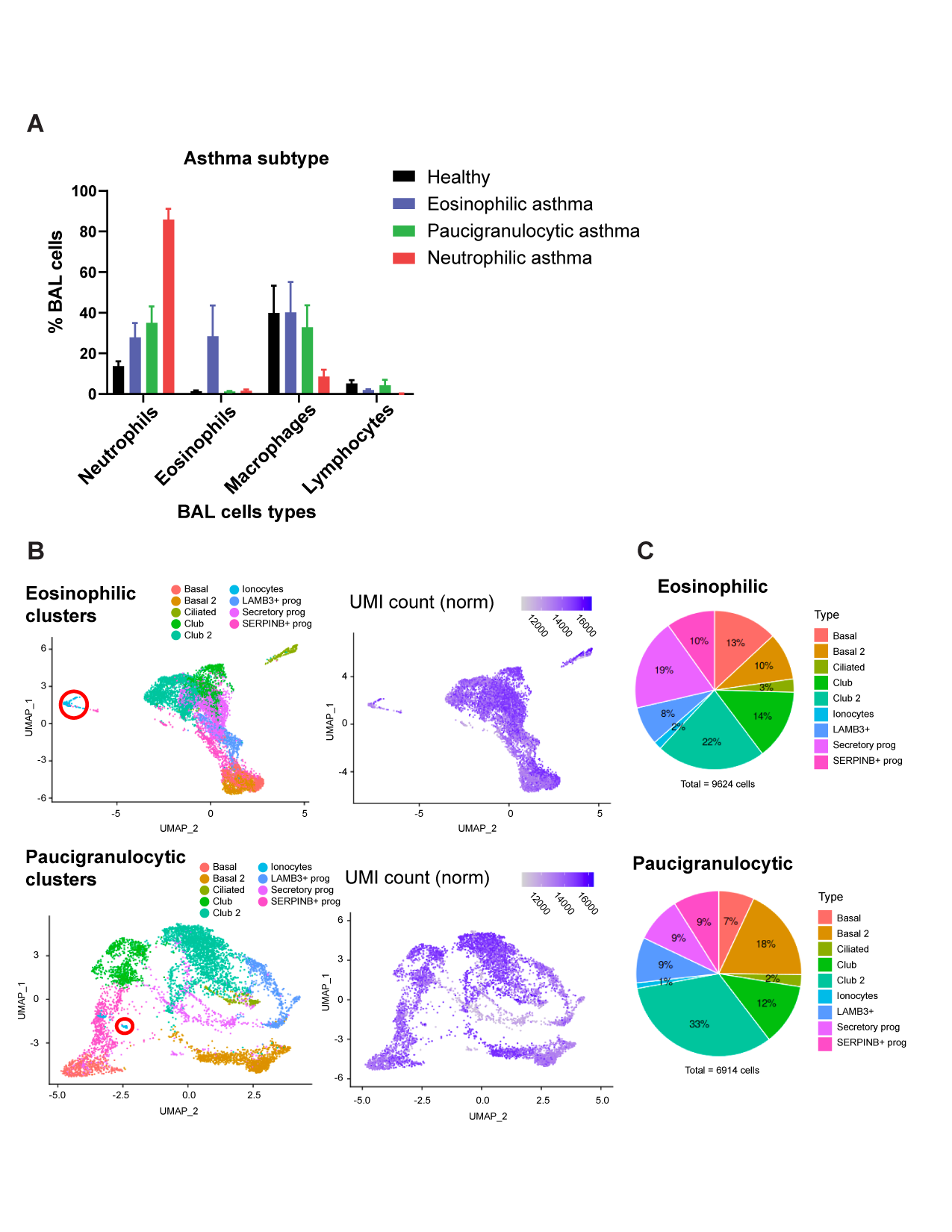

Supplement: Supplementary file 1 — Figure S1. Characterization of patients with severe asthma subtypes and single‐cell RNA‐seq analysis. (A) Percentage from differential cell counts in BAL fluid from healthy subjects and asthma patients subtyped. Single‐cell RNA‐seq was performed on single‐cell suspensions generated from hBECs of eosinophilic asthma patients and paucigranulocytic asthma patients (n = 4 each). (B) Cells were clustered by using a graph‐based shared nearest neighbour method and plotted by UMAP together with heatmaps of gene UMI counts. Nine clusters of cells were identified in all samples and then known cell types were classified using classical gene markers enriched in the clusters through leadingEdge. Transitory progenitors or non‐classified cell types were labelled by predominant transcriptional signatures SERPINB+ prog, LAMB3+ prog or Undefined. (C) The proportion of each cell type in eosinophilic (9624 cells) and paucigranulocytic asthma (6914 cells) patients were calculated using pie charts. [file RESP-30-25-s002.tif]

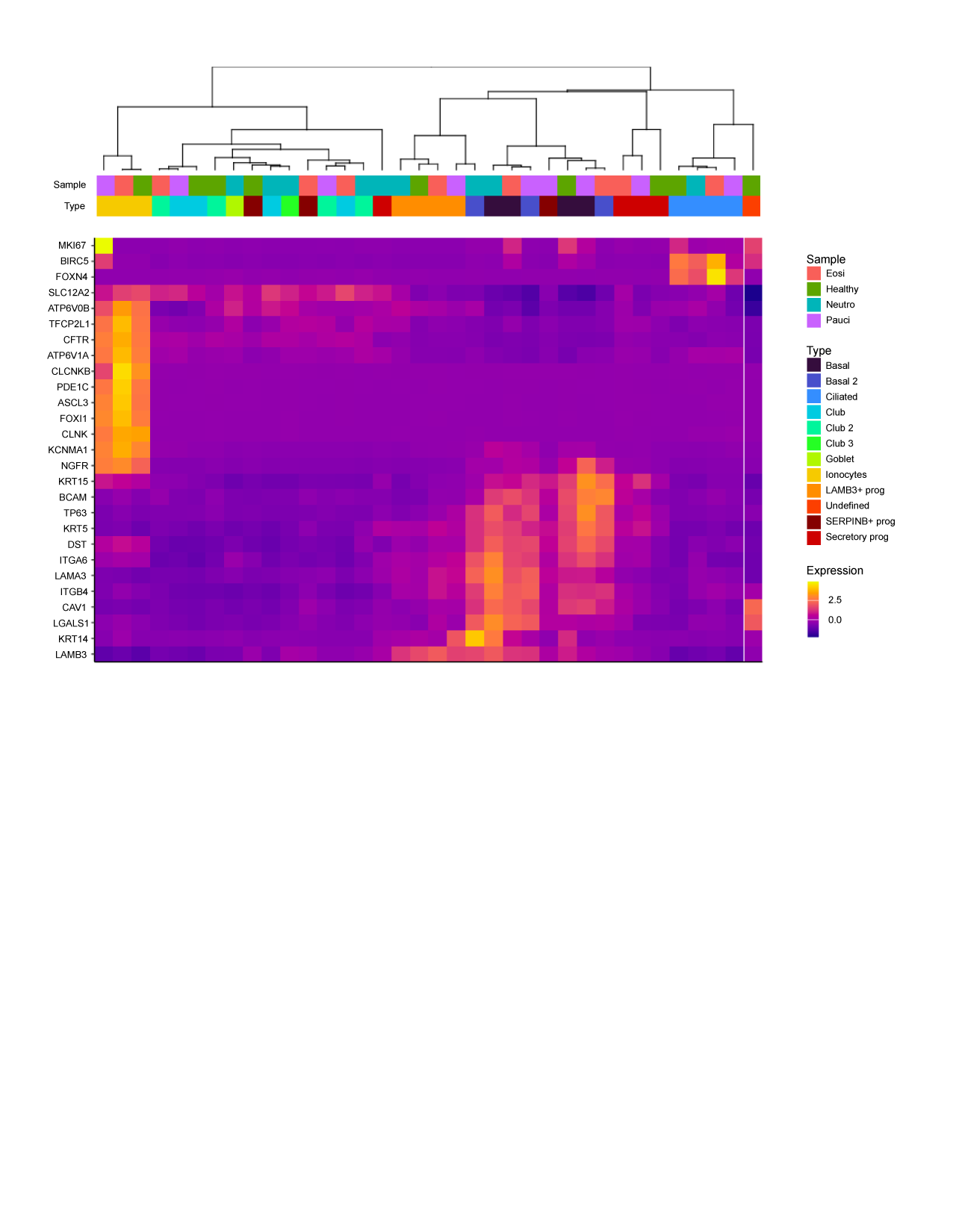

Supplement: Supplementary file 2 — Figure S2. Unsupervised hierarchical clustering of all patient samples and cell subtypes (columns) by known ionocyte and basal cell marker genes (rows), visualized using a heatmap plot. [file RESP-30-25-s004.tif]

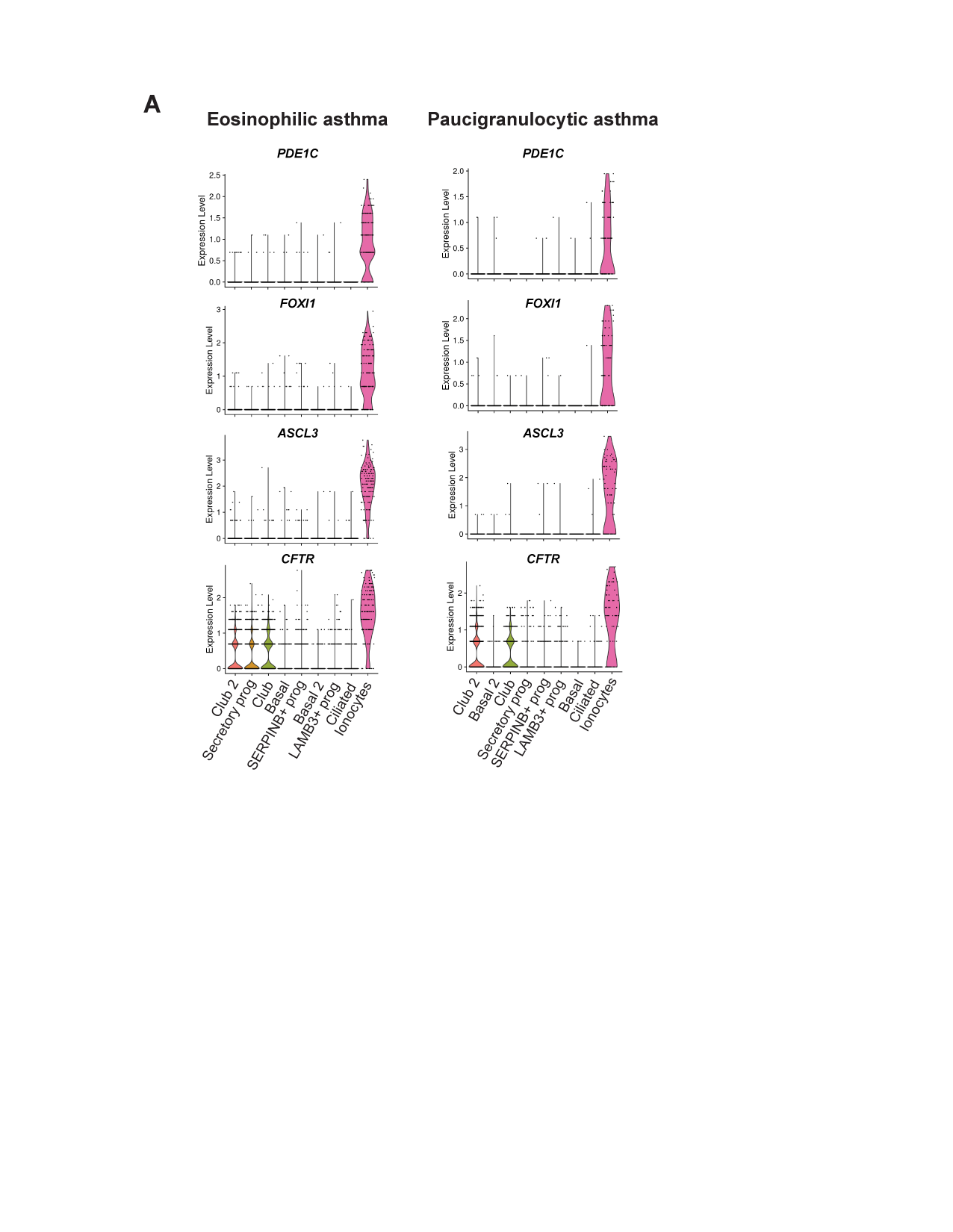

Supplement: Supplementary file 3 — Figure S3. (A) Violin plots of expression of ionocyte gene markers (PDE1C, FOXI1, ASLC3 and CFTR) in each of the 9 cell subtype clusters from eosinophilic versus paucigranulocytic asthma patients. [file RESP-30-25-s009.tif]

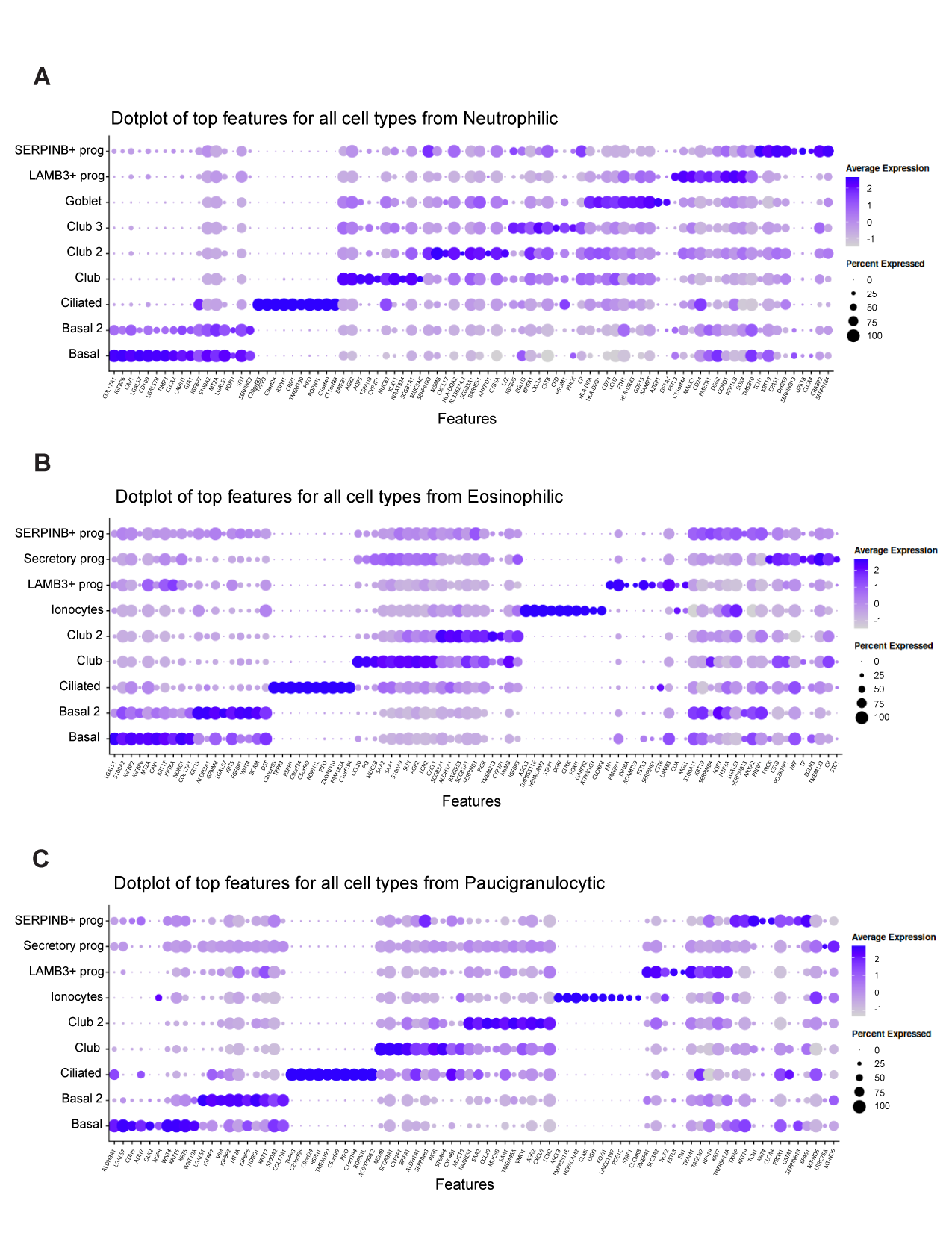

Supplement: Supplementary file 4 — Figure S4. Weighted dot plot showing top 10 features of each cluster from the scRNAseq data derived from cells of (A) neutrophilic, (B) eosinophilic and (C) paucigranulocytic asthma patients. Each dot is sized to represent the percent of cells in each cluster expressing the corresponding top 10 gene, and colours represent the average expression of each maker gene across within that cluster. [file RESP-30-25-s003.tif]

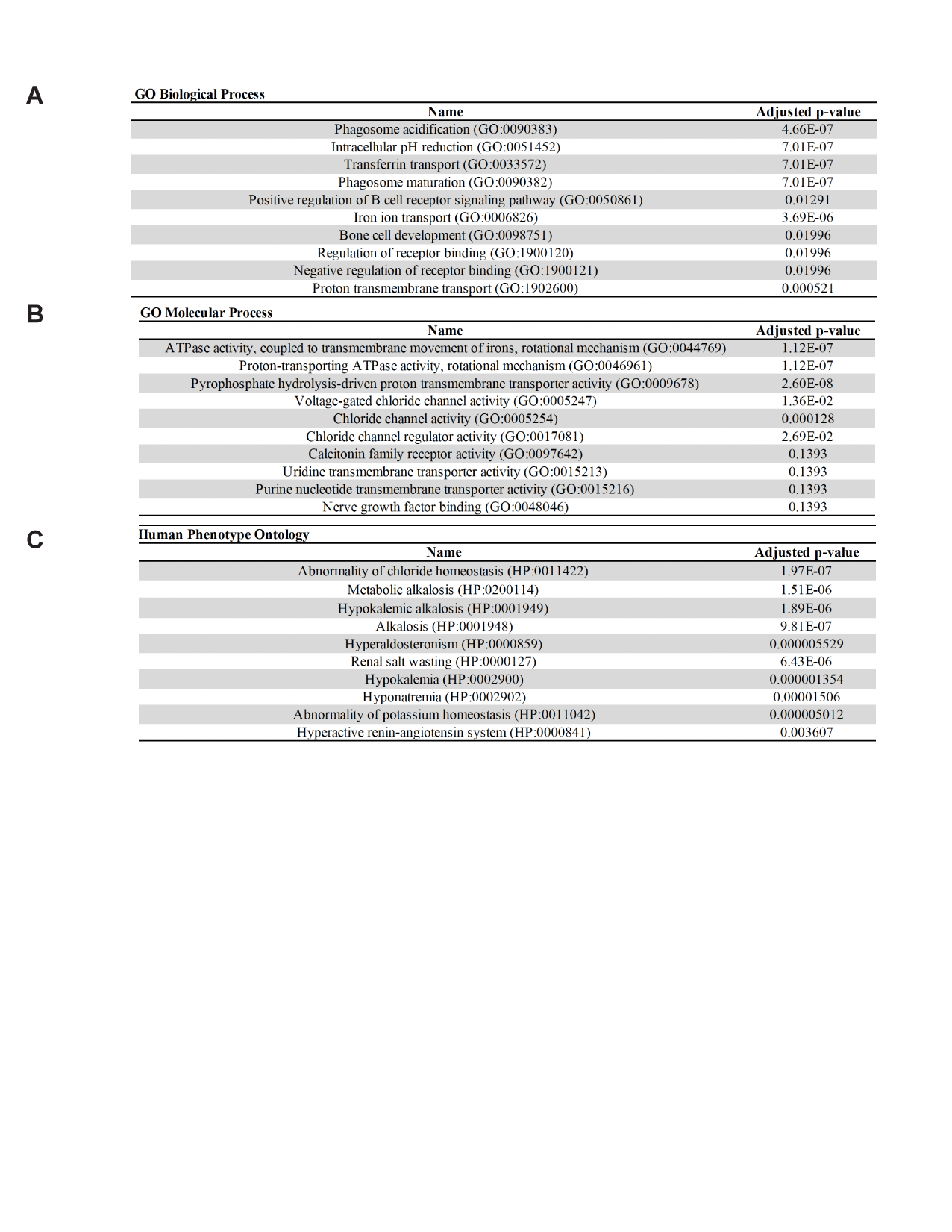

Supplement: Supplementary file 5 — Figure S5. Pathway over‐representation analysis. (A) Gene Ontology (GO) analysis for Biological Process and (B) Molecular Process of the 73 common bronchial ionocyte genes. (C) Human Phenotype Ontology analysis of the 73 common bronchial ionocyte genes. [file RESP-30-25-s001.tif]

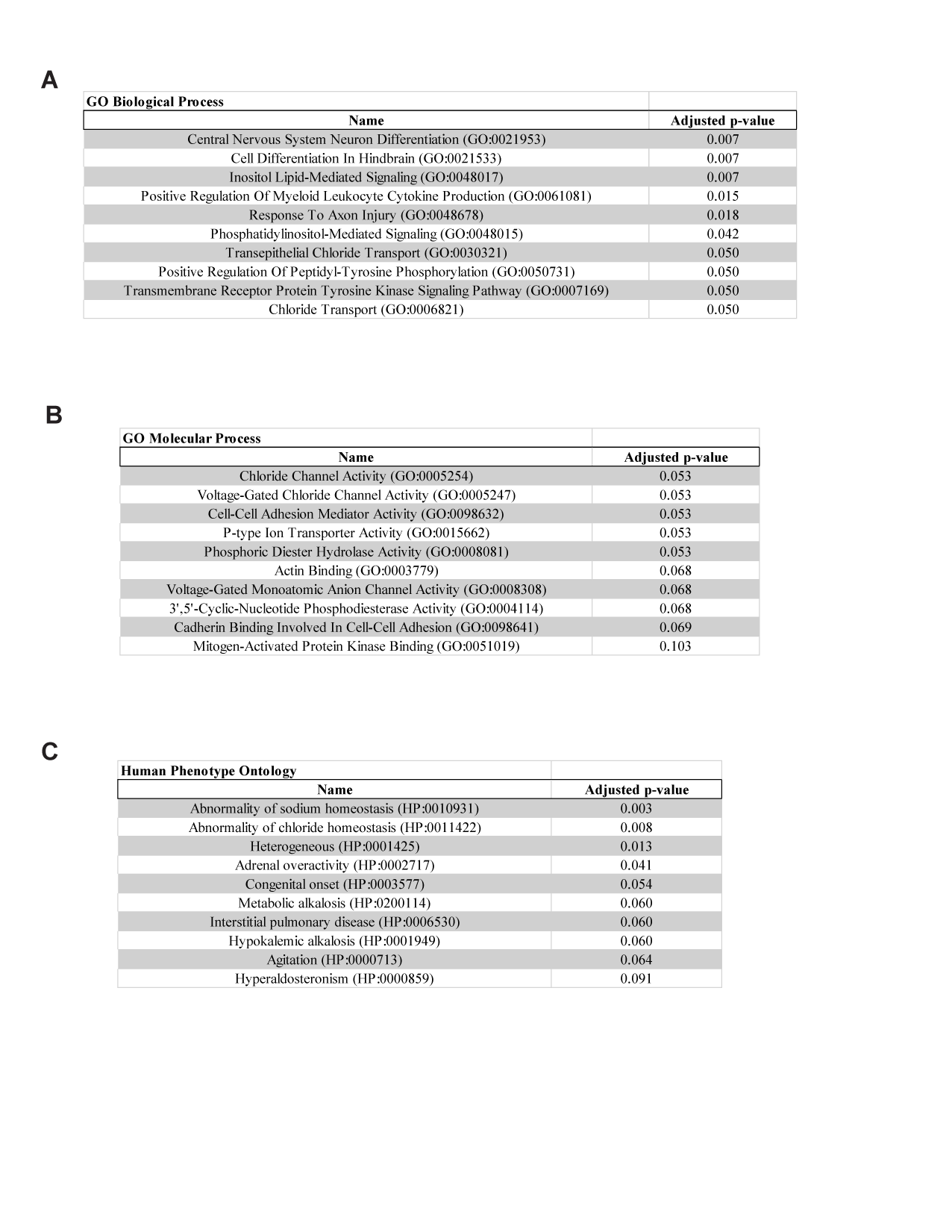

Supplement: Supplementary file 6 — Figure S6. Pathway over‐representation analysis. (A) Gene Ontology (GO) analysis for Biological Process and (B) Molecular Process of the top 100 tracheal ionocyte genes. (C) Human Phenotype Ontology analysis of the top 100 tracheal ionocyte genes. [file RESP-30-25-s006.tif]

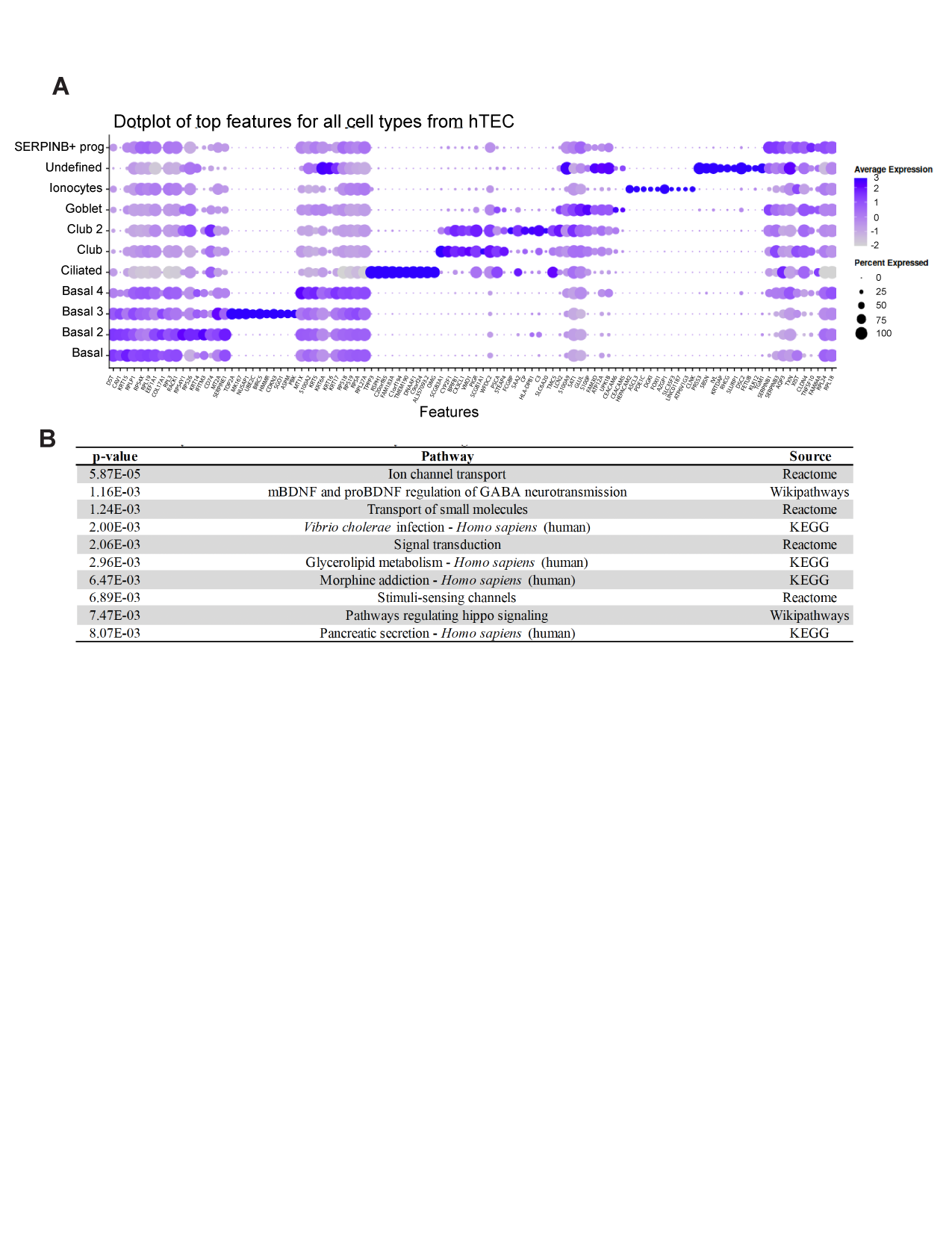

Supplement: Supplementary file 7 — Figure S7. (A) Weighted dot plot showing top 10 features of each cluster from the scRNAseq data derived from human tracheal epithelial cells (hTECs) from healthy donors. Each dot is sized to represent the percent of cells in each cluster expressing the corresponding top 10 genes, and colours represent the average expression of each maker gene across within that cluster. (B) Pathway over‐representation analysis using ConsensusPathDB of the 30 common genes in the ionocyte gene signature from Figure 4D conserved between hBECs of healthy and eosinophilic asthma donors as well as hTECs. [file RESP-30-25-s012.tif]

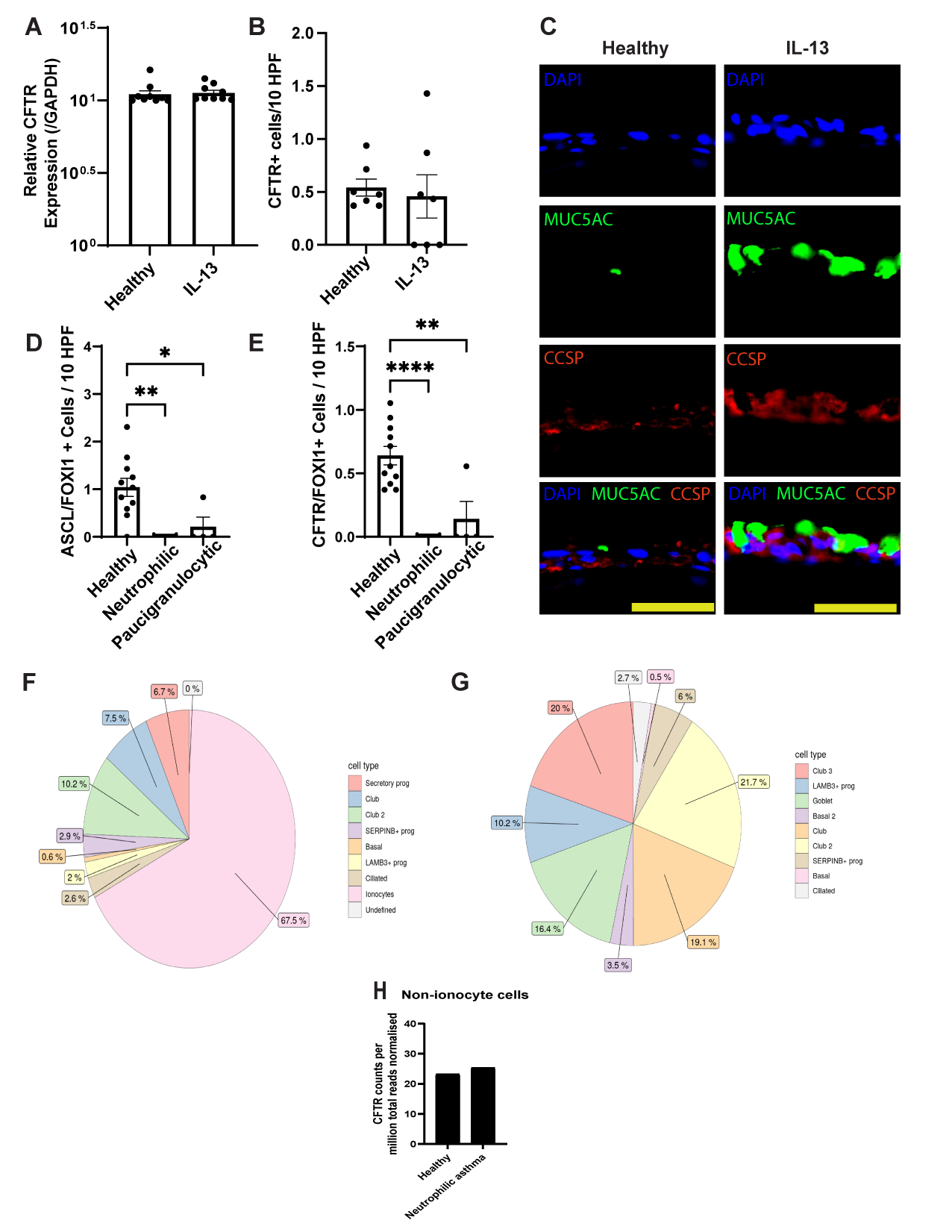

Supplement: Supplementary file 8 — Figure S8. Loss of ionocytes in non‐eosinophilic (neutrophilic and paucigranulocytic) asthma hBECs. (A) CFTR gene expression and (B) quantification of the number of CFTR protein expressing (CFTR+) cells in healthy hBECs treated with or without IL‐13 (n = 7–9). (C) Representative immunofluorescent images of healthy hBECs treated with or without IL‐13 showing increased numbers of CCSP+ and MUC5AC+ goblet cells. (D) ASCL3 + FOXI1+ and (E) CFTR + FOXI1+ in hBECs from neutrophilic (n = 6) and paucigranulocytic (n = 4) asthma or healthy donors (n = 11), values expressed as mean ± SEM. (F‐G) Pie chart relative distribution of CFTR transcript level in each cell type scaled by cluster size for healthy donors (F) or neutrophilic asthma (G). (H) CFTR transcript level in non‐ioncytes normalized by library size across samples as CFTR counts per million total reads. *p ≤ 0.05, **p < 0.01 and ****p < 0.0001. [file RESP-30-25-s008.tif]

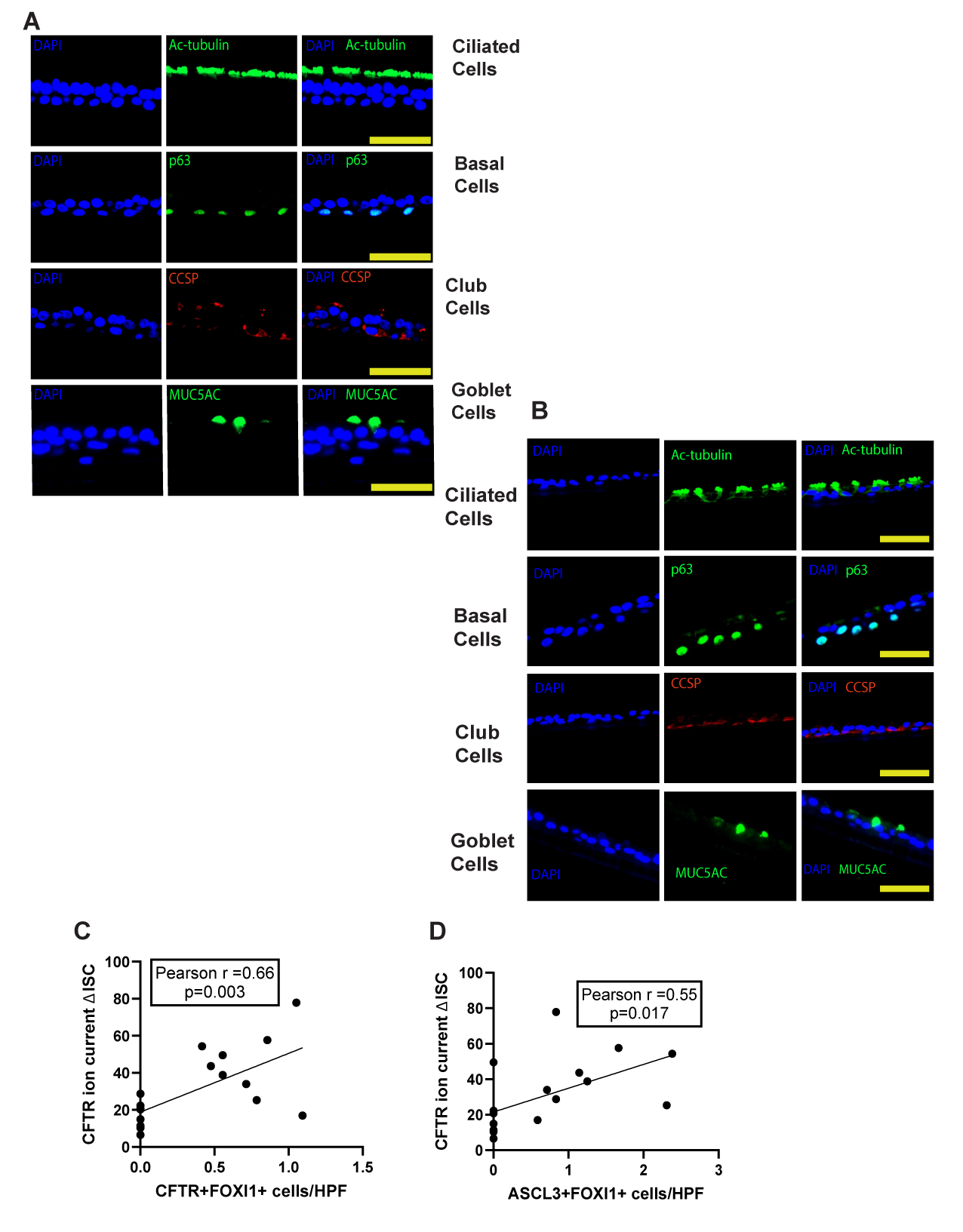

Supplement: Supplementary file 9 — Figure S9. (A, B). Representative immunofluorescent images of healthy (A) or non‐eosinophilic asthma hBECs (B) indicating cells positive for p63 (green), MUC5AC (green), Ac‐tubulin, p63 (green) or CCSP (red), scale bar is 20 μm. (C and D) Linear regression analysis with Pearson r correlation between changes in CFTR ion currents (Ussing chamber) and number of ionocytes per HPF (immunofluorescence). [file RESP-30-25-s011.tif]

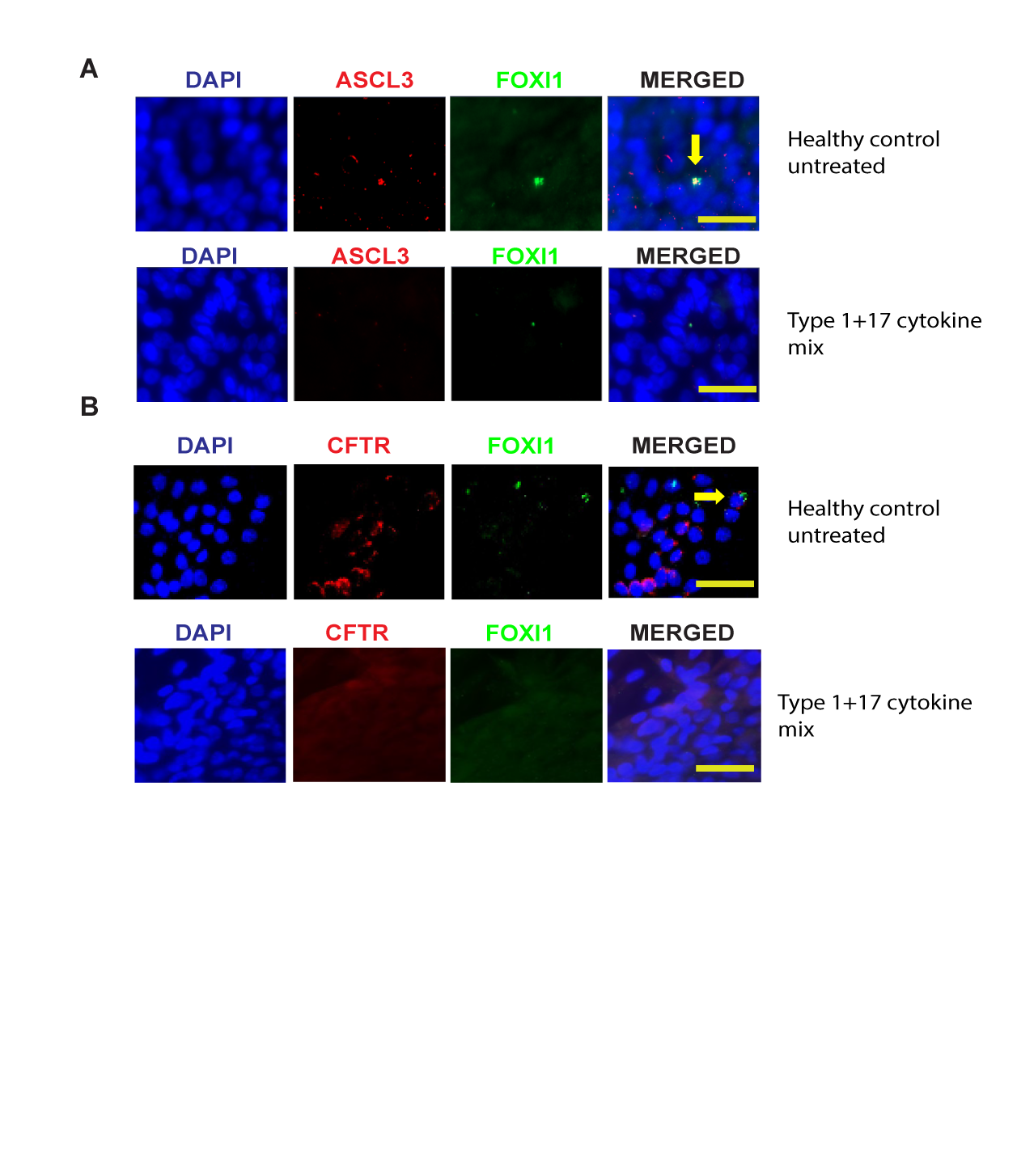

Supplement: Supplementary file 10 — Figure S10. Representative immunofluorescent images of healthy hBECs treated with or without Type 1 + 17 cytokine (IFN‐γ, IL‐17A, TNF‐α and IL‐22) mix showing (A) ASCL3 + FOXI1+ and (B) CFTR + FOXI1+ in hBECs from healthy donors. [file RESP-30-25-s005.tif]

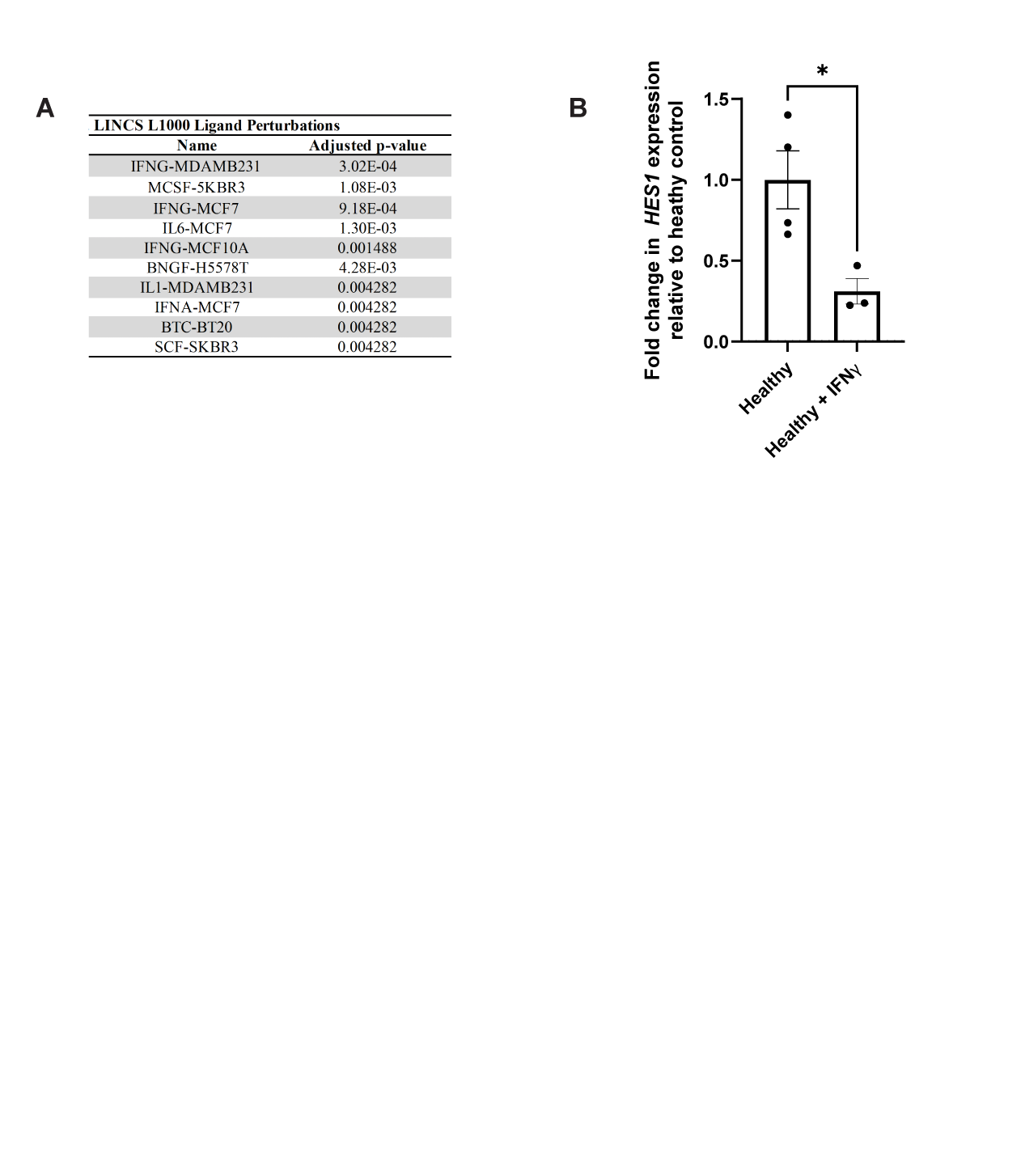

Supplement: Supplementary file 11 — Figure S11. (A) Library of Integrated Network‐Based Cellular Signatures (LINCS) L1000 ligand perturbations analysis showing cytokines and ligands most highly predicted to regulate the ionocyte gene signature. (B) HES1 gene expression measured by qPCR normalized to GAPDH house‐keeper in healthy control hBECs treated with or without IFN‐γ. [file RESP-30-25-s010.tif]

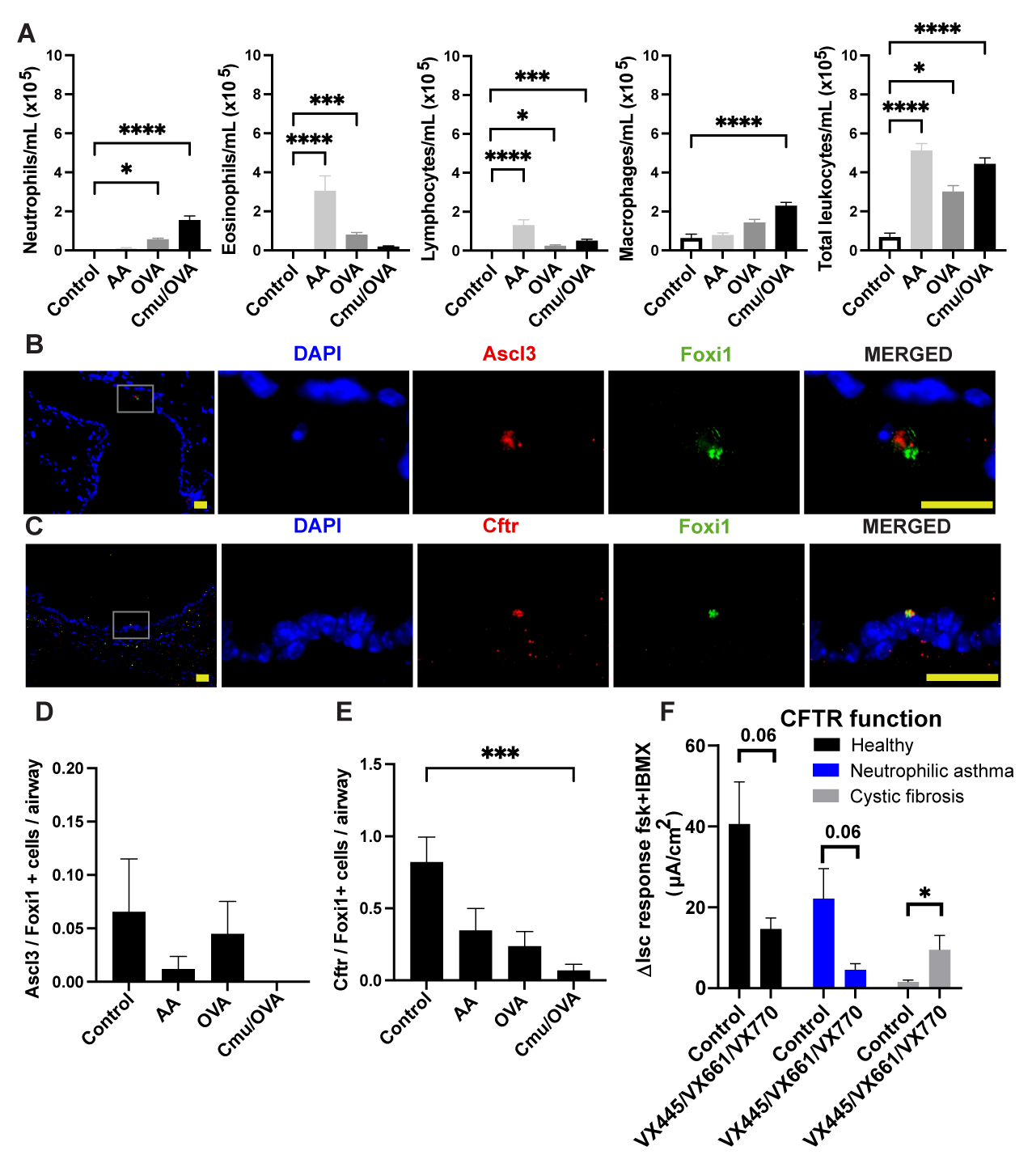

Supplement: Supplementary file 12 — Figure S12. Loss of ionocytes in vivo in a murine model of neutrophilic asthma and impact of CFTR modulators on CFTR function in neutrophilic asthma hBECs. (A) Number of neutrophils, eosinophils, lymphocytes, macrophages and total number of cells/mL in bronchoalveolar lavage (BAL) fluid in control, fungal allergen Alternaria alternata (AA) and protein antigen ovalbumin (OVA) models of T2 asthma, as well as the Chlamydia muridarum (Cmu)/OVA‐treated model of non‐eosinophilic asthma. (B and C) Representative immunofluorescent images of PBS control mouse airways stained with (B) Ascl3 plus Foxi1 or (C) Cftr plus Foxi1, scale bar is 20 μm. (D) Dual Ascl3 + Foxi1+ cells or (E) Cftr + Foxi1+ cells were quantified by immunofluorescence in the bronchi of mice from these asthma models (n = 7–11 mice per group). Values expressed as per airway, mean ± SEM. Kruskal–Wallis test with Dunn's multiple comparison test compared to control mice, *p < 0.05, ***p < 0.001 and ****p < 0.0001. (F) Airway epithelial cells cultured at ALI derived from healthy donors (n = 5), neutrophilic asthma (n = 5) or patients with cystic fibrosis genotype (homozygous F508del/F508del n = 6), were treated with control or the CFTR modulator combination VX445/VX661/VX770, data shows the delta values of short circuit currents (∆Isc) for CFTR function Forskolin + 3‐isobutyl‐1‐methylxanthine (IBMX) stimulated. Values expressed as per airway, mean ± SEM. Wilcoxon paired t test *p < 0.05. [file RESP-30-25-s007.tif]
