# Supplementary material for: Inflammation‐induced loss of CFTR‐expressing airway ionocytes in non‐eosinophilic asthma
Source: Respirology. 2024 Oct 2;30(1):25–40. doi: 10.1111/resp.14833 (PMC11688627; doi:10.1111/resp.14833)
Supplement: Supplementary file 13 — Data S1: Supporting Information. [file RESP-30-25-s013.docx]

**Supplementary Information**

**Appendix 1**

**Methods**

**scRNAseq computational data analysis**

Statistical hypothesis testing with the exception of the likelihood-ratio test (LRT), which is one-tailed, were two-tailed, and exact p-values are reported, except where below the threshold of numerical precision (2.22×10−16). Pre-processing of 3’ droplet-based scRNA-seq data demultiplexing, alignment to the hg38 transcriptome and UMI-collapsing were performed using the CellRanger toolkit (version 3.0.2, 10X Genomics). The CellRanger workflow performs the sample tag and UMI-demultiplexing, reference genome mapping, thresholding and low-quality filtering, clustering and preliminary differential expression analysis. Briefly, sequencer BCL files are demultiplexed to samples by bcl2fastq. Initial inspection and quality control was performed on the CellRanger output. Overall information on sequencing and mapping per sample is listed in Table II. Subsequent data analysis was performed in R (v4.0.2, v4.1.2 (1) and Bioconductor Biobase v2.54 (2) using packages: Seurat v4.0.5 (3), sctransform v0.3.2 (4), uwot v0.1.11 (5, 6), Rtsne v0.15 (7, 8) fgsea (9), gage v2.44 (10).

The criteria used to detect a genes expressed in a cell is dynamically adjusted by CellRanger based on the overall profile of UMI counts per cell, the chemistry version used and the background read profile. For cells with higher than 30% of total cell reads in mitochondrial genes or with less than 250 genes per cell detected, these cells were excluded from downstream analysis. In each sample, data normalisation was performed by a regularised negative binomial regression adjusted by the (residual) percentage of reads in mitochondrial genes, using the SCTransform function in Seurat (4). Then, PCA, UMAP and tSNE projections were performed and the 20 nearest neighbours on the 10 major principal components were computed. An iterative clustering was performed on the kNN data adjusting the resolution parameter to obtain a predetermined number of cell clusters on the dataset, using the simple modularity optimisation graph-based shared nearest neighbour method (11).

Differential gene expression analysis was performed on the regularised pseudo log count data using a Wilcoxon ranked sum analysis on each cell cluster versus all others, identifying genes overexpressed in the cluster of interest. Detected clusters were mapped to known cell types using known overexpressed gene markers (Table III) by leading edge gene set enrichment analysis with an adjusted p value of 0.05 (9). The cluster cell type identification was then confirmed to be also the most significant enrichment assignment using GAGE (generally applicable gene set enrichment for pathway analysis) (10). During the preliminary data analysis due to the large proportion of basal and club cells, multiple clusters expressed high levels of markers for these two cell subtypes. This led to the iterative clustering procedure, reducing the resolution to obtain a more meaningful set of cell subtype clusters. Cell subtype signature genes used in the Venn diagram analysis to identify common signatures that were then used in pathway analysis were obtained by using a minimum FDR of 0.05 for adjusted p values. Genes were then ranked by these adjusted p values, and the top 100 ranked genes for each cluster/cell subtype was used in downstream analysis.

**Measurement and analysis of ciliary beat frequency (CBF)**

Beating cilia of ALI cultured hBECs (frequency 3 to 18 Hz) were captured on a Nikon eclipse Ti2 microscope (Nikon, Japan) with Video Savant 4.0 software using a high-speed digital video recorder. High speed video was recorded at a rate of 300 frames per second and captured a minimum of 512 frames. Measurements of median cilia beat frequency were made using the ciliaFA plugin (12), which utilizes a specific fast Fourier transformation (FFT) algorithm to accurately measure ciliary activity across an entire field. CiliaFA was used together with free open-source ImageJ software (ImageJ, US) and Microsoft Excel to perform analyses.

**Immunofluorescence**

Transwell membranes containing hBECs were fixed using 4% paraformaldehyde (PFA) for 15 min at room temperature. For whole mount staining membranes were embedded in HistoGel (Thermo Scientific) and labelled as previously described (13). Sectioned membranes underwent heat-mediated antigen retrieval with sodium citrate buffer pH 6.0. Whole mount membranes or sections were blocked and permeabilized using PBS containing 3 % BSA, 5 % donkey serum and 0.5 % Triton X-100 for 1 hour at room temperature. Additional blocking was applied using Background Sniper (Biocare Medical) for 10mins at room temperature. Cell membranes were incubated overnight at 4°C with the following primary antibodies anti-CFTR (1:100, 5 µg/ml, R&D MAB23031), anti-FOXI1 (1:250, 2 µg/ml, Abcam ab20454), and anti-ASCL3 (1:100, 1 µg/ml, Sigma-Aldrich HPA027032), anti-Mucin 5AC (MUC5AC, 1:500, 2 µg/ml, Abcam ab212636), anti-acetylated tubulin (Ac-tubulin, 1:1000, 1 µg/ml, Sigma-Aldrich T7451), anti-p63 (1:50, 20 µg/ml, Abcam ab735), and anti-uteroglobin (CCSP, 1:100, 10 µg/ml, Thermo Fisher MA5-34625) at 4°C overnight. Alexa Fluor 555 or 488 conjugated secondary antibodies (1:500, 4 µg/ml, ab150106, ab150129, ab150074) were applied in dark at room temperature for one hour, followed by mounting with Fluoromount-G with DAPI (Invitrogen). The number of ionocytes (ASCL3 and FOXI1 or CFTR and FOXI1 double positives) were quantified per high power field (HPF). The length of cilia (Tubulin) was quantified and presented as µm per mm basement membrane (BM). MUC5AC, p63, and CCSP positive cells were quantified and presented as number of cells per mm BM.

**Supplementary Information: Tables**

**Table S1: Patient characteristics**

|  |  |  | **Non-eosinophilic** | **Non-eosinophilic** |  |
| --- | --- | --- | --- | --- | --- |
|  | **Healthy** | **Non-eosinophilic asthma** | **Neutrophilic asthma** | **Paucigranulocytic asthma** | **Eosinophilic asthma** |
| **n** | 12 | 16 | 8 | 8 | 4 |
| **Age (Mean±SD)** | 60±13.6 | 58.8±13.2 | 65.5±11 | 52±11.7 | 48.3±12.7 |
| **BMI (Mean±SD)** | 24.5±3.8 | 28.8±13.7 | 28.7±14.5 | 28.8±12.9 | 31.2±9.9 |
| **% Predicted FVC (Mean±SD)** | 97.9±13.5 | 85±13.5 | 84.3±12.3 | 85.7±14.8 | 76±9.6 |
| **% Predicted FEV1 (Mean±SD)** | 94.3±15 | 67.8±20 **a** | 74.4±17.1 | 61.1±20.5 **b** | 68.5±15 |
| **FEV1/FVC Ratio (Mean±SD)** | 74.2±8.8 | 65.5±14.6 | 68.4±13.5 | 62.2±15.2 | 73.3±16.4 |
| **% neutrophils TCC in BAL (Mean±SD)** | 20.7±17.9 | 53.9.4±29.4 **a** | 79.8±11.3 **c,d,e** | 32.6±16.5 | 27.9±12.3 |
| **% eosinophils TCC in BAL (Mean±SD)** | 0.8±0.8 | 1.8±1 | 2.2±1 | 1.3±0.8 | 28.4±26.2 **f,g,h,i** |
| **ICS dose (Budesonide mcg/day) (Mean±SD)** | 0 | 1475±706 **l** | 1575±701 **c** | 1375±643 **k** | 1025±576 **j** |
| **GINA stage - severe/persistent** | NA | 100% | 100% | 100% | 100% |
| **CAT1 + CAT2 score** | NA | 3.6±1.7 | 4.6±1.4 **m,n** | 2.5±1.2 | 2.5±1 |
| **FeNO ppB (Mean±SD)** | NA | 29.4±16.8 | 29.8±12.7 | 29±20.5 | 47.5±6.1 |

| *TCC; total cell count* |
| --- |
| *GINA; Global initiative for asthma* |
| *CAT1(cough)+CAT2(mucus) score for chronic bronchitis* |
| *FeNO ppB; fractional exhaled nitric oxide parts per billion* |
| *a = p<0.01 healthy versus non-eosinophilic asthma* |
| *b = p<0.01 healthy versus paucigranulocytic asthma* |
| *c = p<0.0001 healthy versus neutrophilic asthma* |
| *d = p<0.01 eosinophilic versus neutrophilic asthma* |
| *e = p<0.001 paucigranulocytic versus neutrophilic asthma* |
| *f = p<0.0001 healthy versus eosinophilic asthma* |
| *g = p<0.0001 neutrophilic versus eosinophilic asthma* |
| *h = p<0.0001 paucigranulocytic versus eosinophilic asthma* |
| *i = p<0.0001 non-eosinophilic versus eosinophilic asthma* |
| *j = p<0.05 healthy versus eosinophilic asthma* |
| *k = p<0.001 healthy versus paucigranulocytic asthma* |
| *l = p<0.0001 healthy versus non-eosinophilic asthma* |
| *m = p<0.05 eosinophilic versus neutrophilic asthma* |
| *n = p<0.05 paucigranulocytic versus neutrophilic asthma* |

**Table S2: Sequencing and alignment parameters for scRNAseq**

| Sample | Eosinophilic | Neutrophilic | Paucigranulocytic | Healthy |
| --- | --- | --- | --- | --- |
| Reads (MM) | 398 | 285 | 259 | 283 |
| Mapping | 98% | 97% | 97% | 98% |
| In Exons | 74% | 77% | 73% | 73% |
| Valid Barcodes | 97.30% | 98% | 97% | 97% |
| Genes / Cell, median | 3602 | 3983 | 3623 | 3410 |

**Table S3: Known airway epithelial cell subtype markers used in scRNAseq classifications**

| **Basal cells:** TP63, MKI67, NGFR, KRT5, ITGA6, CAV1, BIRC5, KRT14, LGALS1, ITGB4, LAMA3, LAMB3, KRT15, NPPC, BCAM, DST |
| --- |
| **Ciliated cells:** FOXJ1, CDHR3, TUBA1A, TUBB4B, SPAG1, LRRC6, DNAAF1, APOD, CCDC17, CCDC39, SEC14L3, APPL2, TMEM212, FAM161A, CCDC153 |
| **Goblet cells:** MUC5AC, SPDEF, FOXA3, MUC5B, SCGB3A1, DUSP4, MUC20, MUC4, GALNT5, GALNT6, MUC16, TFF2, MSLN, GP2, LIPF |
| **Club cells:** SCGB1A1, CYP2F1, SCGB3A1, MUC5B, TFF3, BPIFB1, BPIFA1, MSMB, SLPI, WFDC2, FOXM1, SFTPD, SYT2, SFTPA1, ERN1, MMP28, CYP4B1, SCGB3A2, CTSE, CYP2E1 |
| **Ionocytes:** CFTR, ASCL3, FOXI1, KCNMA1, CLNK, TFCP2L1, ATP6V1A, ATP6V0B, PDE1C, FOXN4, SLC12A2, CLCNKB |
| **Tuft cells:** GNB3, GNG13, TRPM5, RGS13, GNAT3, PLCB2, TAS1R1 |
| **Neuroendocrine cells:** CGRP, ASCL1, CCK |

**Table S4: Top 100 ionocyte marker genes**

| **Healthy hBEC Ionocyte gene signature top 100 by p value** | |  | **Eosinophilic asthma hBEC Ionocyte gene signature top 100 by p value** | |  | **Healthy hTEC Ionocyte gene signature top 100 by p value** | |
| --- | --- | --- | --- | --- | --- | --- | --- |
| **gene** | **p_val_adj** |  | **gene** | **p_val_adj** |  | **gene** | **p_val_adj** |
| TMPRSS11E | 0 |  | ASCL3 | 0 |  | HEPACAM2 | 1.77e-253 |
| ASCL3 | 0 |  | TMPRSS11E | 0 |  | ASCL3 | 7.42e-235 |
| CLNK | 0 |  | HEPACAM2 | 0 |  | PDE1C | 4.85e-179 |
| HEPACAM2 | 0 |  | STAP1 | 0 |  | DGKI | 1.82e-175 |
| FOXI1 | 0 |  | DGKI | 0 |  | FOXI1 | 1.82e-175 |
| LINC01187 | 0 |  | CLNK | 0 |  | AZGP1 | 3.94e-149 |
| DGKI | 0 |  | FOXI1 | 0 |  | SLC35F3 | 8.48e-146 |
| STAP1 | 0 |  | GABRB2 | 0 |  | LINC01187 | 8.48e-146 |
| CLCNKB | 0 |  | ATP6V1G3 | 0 |  | ATP6V1G3 | 8.48e-146 |
| PDE1C | 0 |  | CLCNKB | 0 |  | CLNK | 9.63e-109 |
| BSND | 0 |  | PDE1C | 0 |  | KIT | 5.91e-102 |
| ATP6V0D2 | 0 |  | NCALD | 0 |  | IGF1 | 3.25e-92 |
| POSTN | 0 |  | TBC1D4 | 0 |  | CLCNKB | 3.55e-92 |
| SLC14A1 | 0 |  | LINC01187 | 0 |  | PLCL1 | 3.71e-92 |
| RCAN2 | 0 |  | KIT | 0 |  | ATP2A3 | 2.38e-91 |
| SLC35F3 | 0 |  | TPX2 | 0 |  | PROX1 | 5.17e-71 |
| RAMP2 | 0 |  | AMACR | 0 |  | ADGRF5 | 7.62e-71 |
| CLCNKA | 0 |  | MARCH10 | 0 |  | TFF3 | 8.39e-60 |
| KIT | 1.60E-303 |  | ANK2 | 0 |  | NEURL1 | 3.41e-56 |
| KCNMA1 | 7.36E-292 |  | BSND | 0 |  | ARNT2 | 1.64e-53 |
| HHIP-AS1 | 4.28E-284 |  | ATP6V0D2 | 0 |  | CLCNKA | 1.63e-50 |
| NEURL1 | 9.14E-272 |  | PROX1 | 0 |  | GABRB2 | 1.19e-49 |
| ANK2 | 7.71E-269 |  | SLC35F3 | 0 |  | PDE11A | 1.80e-49 |
| C11orf16 | 5.07E-264 |  | RCAN2 | 0 |  | CDA | 7.67e-43 |
| FAT3 | 1.32E-246 |  | PTGER3 | 0 |  | NREP | 1.11e-37 |
| DMRT2 | 1.74E-243 |  | CLCNKA | 0 |  | JAM3 | 3.01e-33 |
| NCALD | 8.60E-243 |  | NEURL1 | 0 |  | GPRC5B | 3.85e-33 |
| AMACR | 2.97E-227 |  | SLC14A1 | 0 |  | HES6 | 7.17e-32 |
| CDA | 1.35E-202 |  | DDX4 | 0 |  | PHLDA1 | 5.00e-31 |
| TMEM61 | 5.22E-181 |  | ADCY5 | 0 |  | SULT1C2 | 2.82e-30 |
| ATP2A3 | 7.29E-179 |  | PLCL1 | 0 |  | CCDC110 | 3.13e-30 |
| TBC1D4 | 7.20E-154 |  | SCUBE2 | 0 |  | ITIH5 | 3.13e-30 |
| TFCP2L1 | 1.40E-151 |  | KCNH1 | 0 |  | QPCT | 2.44e-24 |
| MFSD6L | 1.61E-147 |  | HHIP-AS1 | 4.32E-300 |  | RARRES2 | 4.75e-24 |
| SULT1C2 | 4.51E-147 |  | RAMP2 | 1.33E-278 |  | CRYM | 9.19e-22 |
| GABRB2 | 1.01E-139 |  | POSTN | 4.25E-262 |  | C2CD4A | 1.92e-21 |
| NGFR | 1.61E-138 |  | JAM3 | 8.35E-262 |  | HCK | 8.67e-21 |
| MARCH10 | 6.64E-134 |  | FAT3 | 1.10E-250 |  | PTPRJ | 4.27e-18 |
| CEL | 1.24E-125 |  | KCNMA1 | 4.34E-249 |  | SPOCK1 | 5.42e-18 |
| SDC2 | 2.36E-114 |  | DMRT2 | 4.24E-236 |  | B4GALNT2 | 3.38e-17 |
| EFHD1 | 2.48E-98 |  | NCAM1 | 4.81E-234 |  | DUSP10 | 4.90e-17 |
| ADGRF5 | 5.31E-97 |  | LTBP2 | 1.88E-208 |  | DNAJC12 | 7.13e-17 |
| LTBP2 | 1.49E-94 |  | FAM3B | 5.00E-198 |  | CELSR3 | 9.19e-17 |
| RARRES2 | 6.34E-91 |  | SCG2 | 1.23E-191 |  | ABCA3 | 4.46e-16 |
| CD9 | 2.87E-88 |  | C11orf16 | 2.06E-184 |  | CFTR | 6.26e-15 |
| ATP6V1C2 | 2.36E-87 |  | ATP2A3 | 2.11E-173 |  | CORO7 | 1.54e-14 |
| CFTR | 1.21E-86 |  | CEL | 4.05E-172 |  | C11orf16 | 1.83e-14 |
| KRT8 | 1.79E-84 |  | TMEM61 | 1.00E-160 |  | NCALD | 2.72e-14 |
| HSD11B2 | 6.64E-80 |  | SDC2 | 1.66E-156 |  | MARCKSL1 | 6.30e-13 |
| ATP6V0B | 1.23E-78 |  | EFHD1 | 4.42E-156 |  | ANKRD33B | 1.26e-10 |
| AKR1B1 | 2.12E-78 |  | NGFR | 6.63E-153 |  | ESRRG | 2.65e-10 |
| C12orf75 | 2.31E-78 |  | ATP6V1C2 | 1.73E-150 |  | LPGAT1 | 3.82e-10 |
| PLCB4 | 4.80E-78 |  | MFSD6L | 1.55E-139 |  | NOS2 | 5.91e-10 |
| NCKAP5 | 4.67E-77 |  | HSD11B2 | 1.21E-136 |  | DPYSL3 | 9.95e-10 |
| ATP6V1A | 3.74E-72 |  | TFCP2L1 | 1.57E-136 |  | NGFR | 3.43e-9 |
| ITPR2 | 1.55E-69 |  | FAM131C | 3.65E-131 |  | FAT3 | 7.21e-9 |
| SLC29A1 | 2.72E-66 |  | CSGALNACT1 | 2.44E-126 |  | NTHL1 | 1.32e-8 |
| RASSF6 | 5.74E-63 |  | SHISA8 | 2.89E-125 |  | TBC1D1 | 5.32e-8 |
| FOXP1 | 2.17E-62 |  | CFTR | 4.38E-122 |  | ZG16B | 7.21e-8 |
| MT-CO2 | 1.69E-58 |  | CDA | 8.41E-120 |  | FGFR1 | 1.44e-7 |
| IMPA2 | 5.29E-58 |  | ATP6V0A4 | 2.02E-117 |  | BPIFA2 | 4.14e-7 |
| ATP6V0A4 | 1.31E-56 |  | NRIP3 | 5.29E-106 |  | FOXP1 | 4.85e-7 |
| BCL2 | 2.53E-56 |  | SULT1C2 | 2.07E-105 |  | SNTB1 | 6.17e-7 |
| MT-ATP6 | 6.02E-56 |  | PLCB4 | 1.97E-101 |  | FAIM2 | 0.00000111 |
| BAALC | 1.31E-55 |  | ATP7A | 4.72E-99 |  | MARCH10 | 0.00000111 |
| HILPDA | 5.34E-55 |  | SLC29A1 | 3.82E-97 |  | PACRG | 0.00000169 |
| CBR3 | 6.18E-55 |  | SCIN | 1.13E-96 |  | ARRB1 | 0.00000206 |
| MTSS1 | 8.40E-55 |  | TMEM171 | 3.25E-94 |  | SYT17 | 0.00000224 |
| FAM3B | 1.13E-54 |  | ATP6V0B | 6.09E-94 |  | GPSM3 | 0.0000026 |
| PLCG2 | 3.03E-54 |  | CD9 | 2.88E-92 |  | ZBTB10 | 0.00000395 |
| TFF3 | 5.12E-53 |  | ADGRF5 | 4.25E-90 |  | ZNF704 | 0.000004 |
| HES6 | 7.72E-53 |  | SNTB1 | 5.88E-89 |  | AKR1B1 | 0.00000818 |
| ATP6V1F | 1.19E-52 |  | PACRG | 3.69E-87 |  | NRIP3 | 0.0000135 |
| FAM43A | 8.38E-52 |  | C12orf75 | 8.46E-86 |  | IQGAP2 | 0.0000367 |
| PHLDA1 | 7.73E-51 |  | AKR1B1 | 3.02E-85 |  | ATP6V0B | 0.0000415 |
| MT-CO1 | 1.60E-49 |  | ATP6V1A | 8.74E-85 |  | TMPRSS11E | 0.0000436 |
| MT-CYB | 4.75E-49 |  | ID4 | 9.09E-85 |  | PLCG2 | 0.0000453 |
| GOLM1 | 2.13E-48 |  | GOLM1 | 8.38E-79 |  | MCF2L | 0.0000488 |
| IQGAP2 | 8.74E-48 |  | IMPA2 | 4.01E-78 |  | EPCAM | 0.0000625 |
| ATP1A1 | 1.77E-47 |  | RASSF6 | 2.70E-74 |  | FAM117A | 0.0000645 |
| KRT18 | 3.53E-47 |  | CCDC28B | 2.97E-74 |  | TMEM51 | 0.0000687 |
| ST14 | 3.94E-47 |  | FOXP1 | 6.64E-74 |  | FAM43A | 0.000105 |
| RALA | 6.98E-46 |  | OTULINL | 7.44E-72 |  | MAN1C1 | 0.000124 |
| H3F3B | 9.90E-46 |  | ATP6V1F | 1.66E-71 |  | SEMA3C | 0.00013 |
| CRACR2A | 1.49E-45 |  | ATP1A1 | 4.69E-71 |  | MUC5AC | 0.00013 |
| SLC25A5 | 6.99E-45 |  | CORO7 | 3.27E-70 |  | AL162231.1 | 0.000135 |
| SCIN | 1.76E-43 |  | APLP2 | 3.31E-70 |  | ATP7A | 0.000217 |
| CORO7 | 2.91E-43 |  | B2M | 1.69E-69 |  | MXD4 | 0.000223 |
| GNAS | 4.88E-43 |  | MT-CO2 | 6.72E-69 |  | MGAT4A | 0.00024 |
| CALM2 | 9.11E-43 |  | MT-ATP6 | 5.89E-68 |  | ENC1 | 0.000254 |
| ESRRG | 1.15E-42 |  | SCNN1B | 2.41E-65 |  | PBXIP1 | 0.000318 |
| GPRC5B | 2.75E-42 |  | KRT8 | 2.72E-65 |  | CACNA1A | 0.000386 |
| CD24 | 2.76E-42 |  | SEC11C | 3.07E-65 |  | ASNS | 0.0004 |
| TPD52 | 2.89E-42 |  | TPD52 | 2.14E-64 |  | KRT18 | 0.000456 |
| TBC1D1 | 5.60E-42 |  | IQGAP2 | 8.40E-64 |  | FAM174B | 0.000628 |
| MT-CO3 | 1.00E-41 |  | RERG | 1.16E-63 |  | DMRT2 | 0.000806 |
| B2M | 1.35E-41 |  | SLC25A5 | 1.56E-63 |  | FARP1 | 0.00108 |
| APLP2 | 6.39E-41 |  | ID3 | 1.57E-61 |  | PXDN | 0.00122 |
| SCNN1B | 1.35E-40 |  | MT-CO1 | 5.87E-59 |  | NAV1 | 0.00163 |
| ANXA4 | 1.53E-40 |  | SCN9A | 4.09E-56 |  | QSOX1 | 0.00172 |

**Supplementary Information: Figures**

**Figure Legends**

**Figure S1.** Characterisation of patients with severe asthma subtypes and single-cell RNA-seq analysis. (A) Percentage from differential cell counts in BAL fluid from healthy subjects and asthma patients subtyped. Single-cell RNA-seq was performed on single-cell suspensions generated from hBECs of eosinophilic asthma patients and paucigranulocytic asthma patients (n=4 each). (B) Cells were clustered by using a graph-based shared nearest neighbour method and plotted by UMAP together with heatmaps of gene UMI counts. 9 clusters of cells were identified in all samples and then known cell types were classified using classical gene markers enriched in the clusters through leadingEdge. Transitory progenitors or non-classified cell types were labelled by predominant transcriptional signatures SERPINB+ prog, LAMB3+ prog, or Undefined. (C) The proportion of each cell type in eosinophilic (9624 cells) and paucigranulocytic asthma (6914 cells) patients were calculated using pie charts.

**Figure S2.** Unsupervised hierarchical clustering of all patient samples and cell subtypes (columns) by known ionocyte and basal cell marker genes (rows), visualised using a heatmap plot.

**Figure S3.** (A) Violin plots of expression of ionocyte gene markers (*PDE1C, FOXI1, ASLC3* and *CFTR*) in each of the 9 cell subtype clusters from eosinophilic versus paucigranulocytic asthma patients.

**Figure S4.** Weighted dot plot showing top 10 features of each cluster from the scRNAseq data derived from cells of (A) neutrophilic, (B) eosinophilic, and (C) paucigranulocytic asthma patients. Each dot is sized to represent the percent of cells in each cluster expressing the corresponding top 10 gene, and colours represent the average expression of each maker gene across within that cluster.

**Figure S5.** Pathway over-representation analysis. (A) Gene Ontology (GO) analysis for Biological Process and (B) Molecular Process of the 73 common bronchial ionocyte genes. (C) Human Phenotype Ontology analysis of the 73 common bronchial ionocyte genes.

**Figure S6.** Pathway over-representation analysis. (A) Gene Ontology (GO) analysis for Biological Process and (B) Molecular Process of the top 100 tracheal ionocyte genes. (C) Human Phenotype Ontology analysis of the top 100 tracheal ionocyte genes.

**Figure S7.**  (A) Weighted dot plot showing top 10 features of each cluster from the scRNAseq data derived from human tracheal epithelial cells (hTECs) from healthy donors. Each dot is sized to represent the percent of cells in each cluster expressing the corresponding top 10 genes, and colours represent the average expression of each maker gene across within that cluster. (B) Pathway over-representation analysis using ConsensusPathDB of the 30 common genes in the ionocyte gene signature from Fig 4D conserved between hBECs of healthy and eosinophilic asthma donors as well as hTECs.

**Figure S8.** Loss of ionocytes in non-eosinophilic (neutrophilic and paucigranulocytic) asthma hBECs. (A) *CFTR* gene expression, and (B) quantification of the number of CFTR protein expressing (CFTR+) cells in healthy hBECs treated with or without IL-13 (n=7-9). (C) Representative immunofluorescent images of healthy hBECs treated with or without IL-13 showing increased numbers of CCSP+ and MUC5AC+ goblet cells. (D) ASCL3+FOXI1+ and (E) CFTR+FOXI1+ in hBECs from neutrophilic (n=6) and paucigranulocytic (n=4) asthma or healthy donors (n=11), values expressed as mean ± SEM. (F-G) Pie chart relative distribution of CFTR transcript level in each cell type scaled by cluster size for healthy donors (F) or neutrophilic asthma (G). (H) CFTR transcript level in non-ioncytes normalised by library size across samples as CFTR counts per million total reads. *p ≤ 0.05, **p < 0.01, ****p < 0.0001.

**Figure S9.** (A-B). Representative immunofluorescent images of healthy (A) or non-eosinophilic asthma hBECs (B) indicating cells positive for p63 (green), MUC5AC (green), Ac-tubulin, p63 (green), or CCSP (red), scale bar is 20μm. (C and D) Linear regression analysis with Pearson r correlation between changes in CFTR ion currents (Ussing chamber) and number of ionocytes per HPF (immunofluorescence).

**Figure S10.** Representative immunofluorescent images of healthy hBECs treated with or without Type 1 + 17 cytokine (IFN-γ, IL-17A, TNF-α, and IL-22) mix showing (A) ASCL3+FOXI1+ and (B) CFTR+FOXI1+ in hBECs from healthy donors.

**Figure S11.** (A) Library of Integrated Network-Based Cellular Signatures (LINCS) L1000 ligand perturbations analysis showing cytokines and ligands most highly predicted to regulate the ionocyte gene signature. (B) *HES1* gene expression measured by qPCR normalised to *GAPDH* house-keeper in healthy control hBECs treated with or without IFN-γ.

**Figure S12.** Loss of ionocytes *in vivo* in a murine model of neutrophilic asthma and impact of CFTR modulators on CFTR function in neutrophilic asthma hBECs. (A) Number of neutrophils, eosinophils, lymphocytes, macrophages, and total number of cells/mL in bronchoalveolar lavage (BAL) fluid in control, fungal allergen *Alternaria alternata* (AA), and protein antigen ovalbumin (OVA) models of T2 asthma, as well as the *Chlamydia* *muridarum* (Cmu)/OVA-treated model of non-eosinophilic asthma. (B and C) Representative immunofluorescent images of PBS control mouse airways stained with (B) Ascl3 plus Foxi1, or (C) Cftr plus Foxi1, scale bar is 20 µm. (D) Dual Ascl3+Foxi1+ cells or (E) Cftr+Foxi1+ cells were quantified by immunofluorescence in the bronchi of mice from these asthma models (n=7-11 mice per group). Values expressed as per airway, mean ± SEM. Kruskal-Wallis test with Dunn’s multiple comparison test compared to control mice, *p<0.05, ***p < 0.001, ****p < 0.0001. (F) Airway epithelial cells cultured at ALI derived from healthy donors (n=5), neutrophilic asthma (n=5), or patients with cystic fibrosis genotype (homozygous F508del/F508del n=6), were treated with control or the CFTR modulator combination VX445/VX661/VX770, data shows the delta values of short circuit currents (∆Isc) for CFTR function Forskolin + 3-isobutyl-1-methylxanthine (IBMX) stimulated. Values expressed as per airway, mean ± SEM. Wilcoxon paired t test *p<0.05.

**References**

1. CoreRTeam. R: A language and environment for statistical computing. Vienna, Austria: R Foundation for Statistical Computing; 2017.

2. Huber W, Carey VJ, Gentleman R, Anders S, Carlson M, Carvalho BS, et al. Orchestrating high-throughput genomic analysis with Bioconductor. Nat Methods. 2015;12(2):115-21.

3. Hao Y, Hao S, Andersen-Nissen E, Mauck WM, 3rd, Zheng S, Butler A, et al. Integrated analysis of multimodal single-cell data. Cell. 2021;184(13):3573-87.e29.

4. Hafemeister C, Satija R. Normalization and variance stabilization of single-cell RNA-seq data using regularized negative binomial regression. Genome Biol. 2019;20(1):296.

5. Becht E, McInnes L, Healy J, Dutertre CA, Kwok IWH, Ng LG, et al. Dimensionality reduction for visualizing single-cell data using UMAP. Nat Biotechnol. 2018.

6. Melville J. uwot: The uniform manifold approximation and projection (UMAP) method for dimensionality reduction: <https://CRAN.R-project.org/> package=uwot; [Available from: <https://CRAN.R-project.org/> package=uwot.

7. van der Maaten L. Accelerating t-SNE using Tree-Based Algorithms. Journal of Machine Learning Research. 2015;15:3221-45.

8. Krijthe J. Rtsne: T-Distributed stochastic neighbor embedding using Barnes-Hut implementation: <https://github.com/jkrijthe/Rtsne>; 2015 [Available from: <https://github.com/jkrijthe/Rtsne>.

9. Korotkevich G, Sukhov V, Budin N, Shpak B, Artyomov MN, Sergushichev A. Fast gene set enrichment analysis. bioRxiv. 2021:060012.

10. Luo W, Friedman MS, Shedden K, Hankenson KD, Woolf PJ. GAGE: generally applicable gene set enrichment for pathway analysis. BMC Bioinformatics. 2009;10:161.

11. Blondel V, Guillaume J-L, Lambiotte R, Lefebvre E. Fast Unfolding of Communities in Large Networks. Journal of Statistical Mechanics Theory and Experiment. 2008;2008.

12. Smith CM, Djakow J, Free RC, Djakow P, Lonnen R, Williams G, et al. ciliaFA: a research tool for automated, high-throughput measurement of ciliary beat frequency using freely available software. Cilia. 2012;1:14.

13. Reid AT, Nichol KS, Chander Veerati P, Moheimani F, Kicic A, Stick SM, et al. Blocking Notch3 Signaling Abolishes MUC5AC Production in Airway Epithelial Cells from Individuals with Asthma. Am J Respir Cell Mol Biol. 2020;62(4):513-23.
